# Supplementary material for: KATP Channel Inhibitors Reduce Cell Proliferation Through Upregulation of H3K27ac in Diffuse Intrinsic Pontine Glioma: A Functional Expression Investigation
Source: Cancers (Basel). 2025 Jan 22;17(3):358. doi: 10.3390/cancers17030358 (PMC11816144; doi:10.3390/cancers17030358)
Supplement: Supplementary file 1 [file cancers-17-00358-s001.zip › Supplementary File 1. (Figures S1-S5 and Table S1 in the manuscript).pptx]

## Slide 1
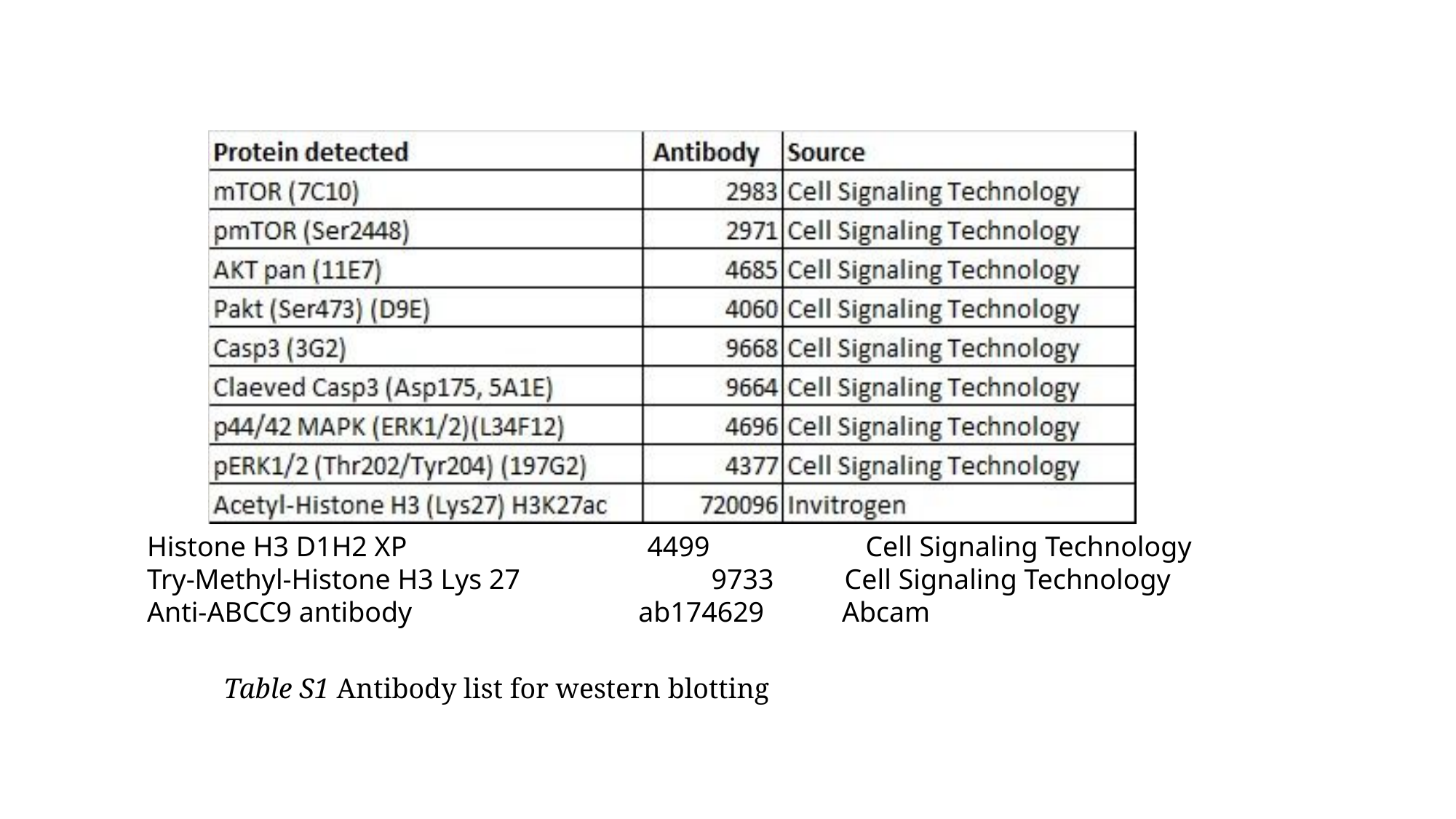

Histone H3 D1H2 XP 		 4499	 Cell Signaling Technology
Try-Methyl-Histone H3 Lys 27 9733 Cell Signaling Technology
Anti-ABCC9 antibody ab174629 Abcam
Table S1 Antibody list for western blotting

## Slide 2
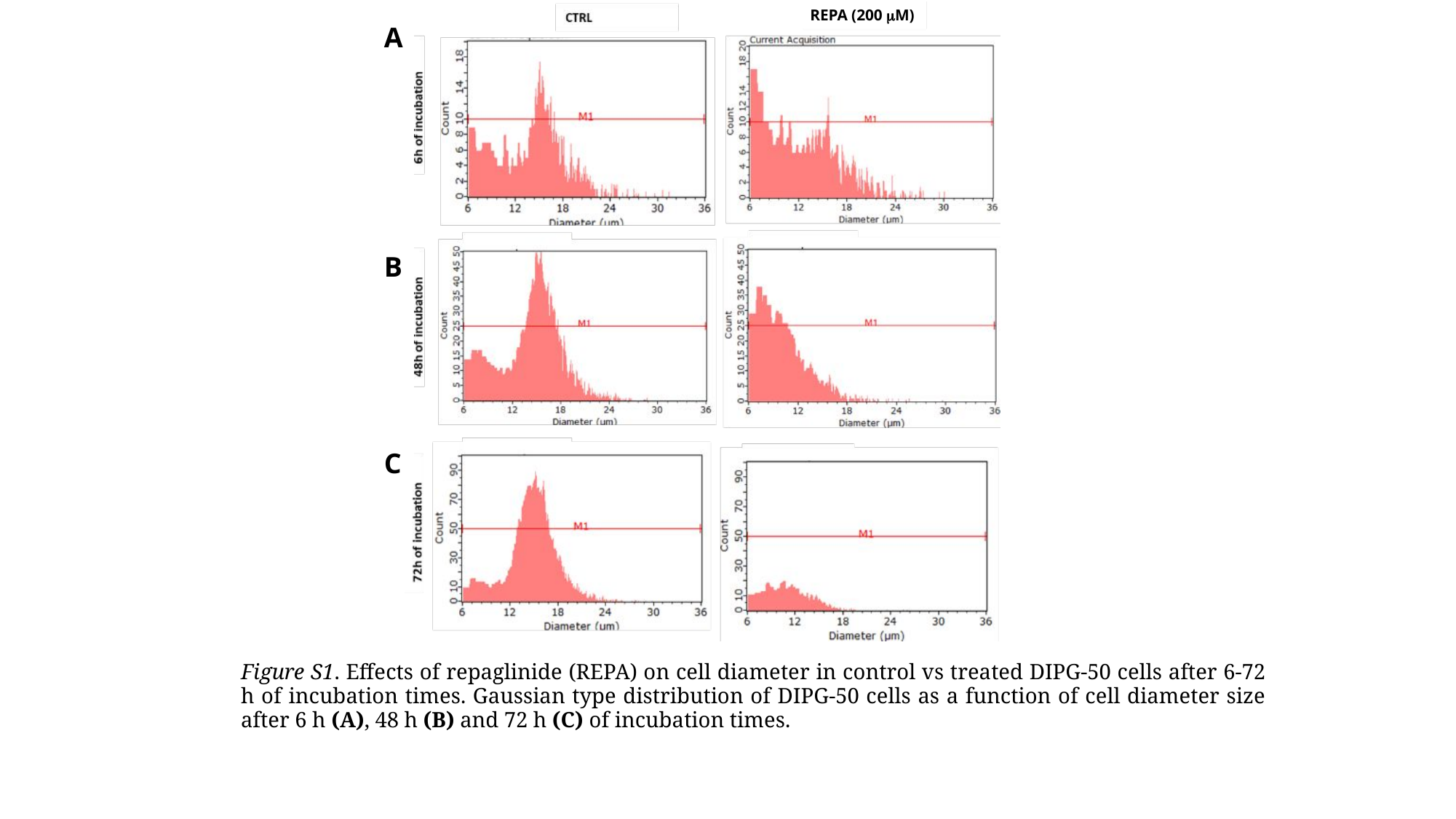

REPA (200 mM)
(a)
(b)
(c)
A
B
C
Figure S1. Effects of repaglinide (REPA) on cell diameter in control vs treated DIPG-50 cells after 6-72 h of incubation times. Gaussian type distribution of DIPG-50 cells as a function of cell diameter size after 6 h (A), 48 h (B) and 72 h (C) of incubation times.

## Slide 3
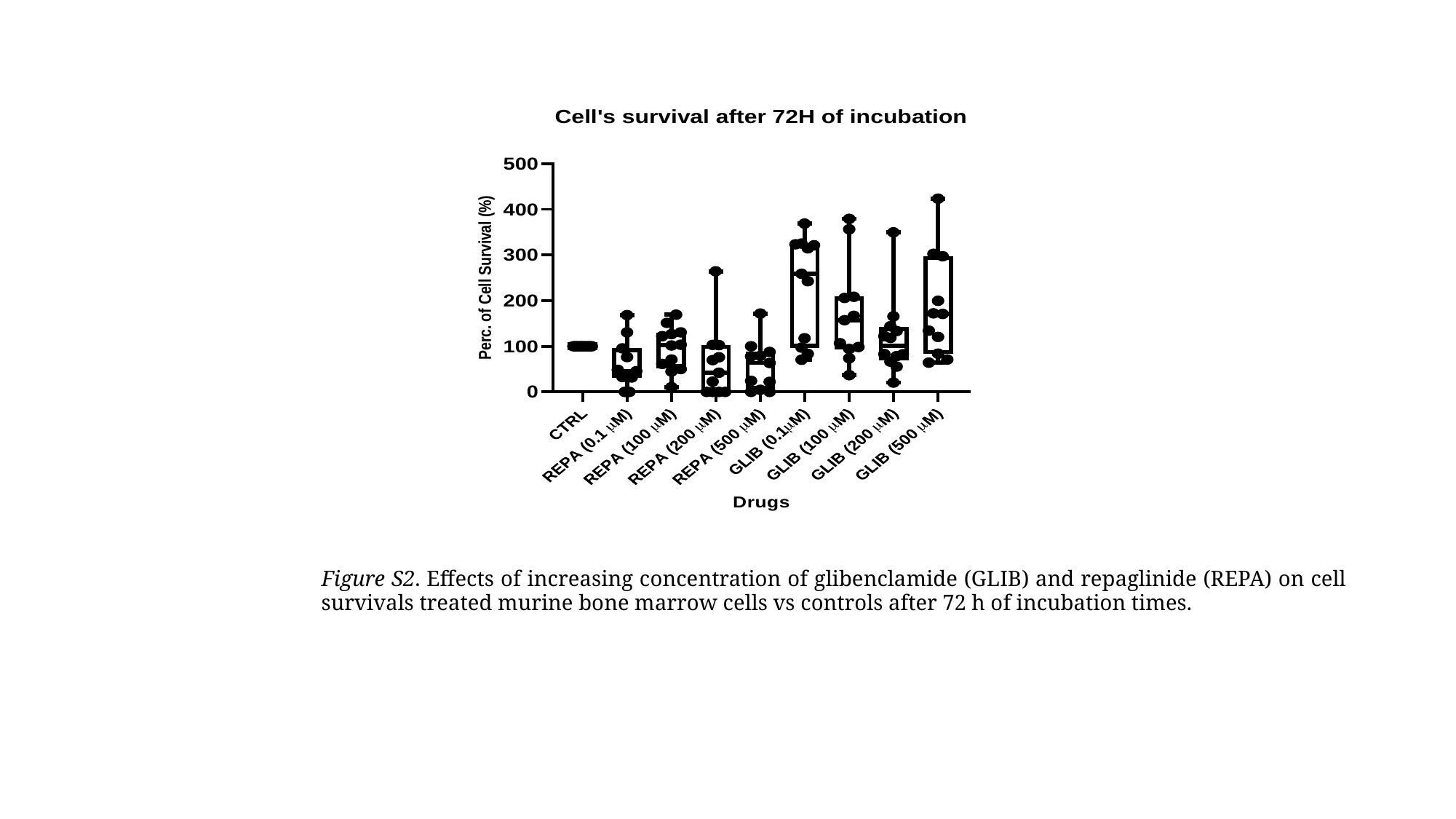

Figure S2. Effects of increasing concentration of glibenclamide (GLIB) and repaglinide (REPA) on cell survivals treated murine bone marrow cells vs controls after 72 h of incubation times.

## Slide 4
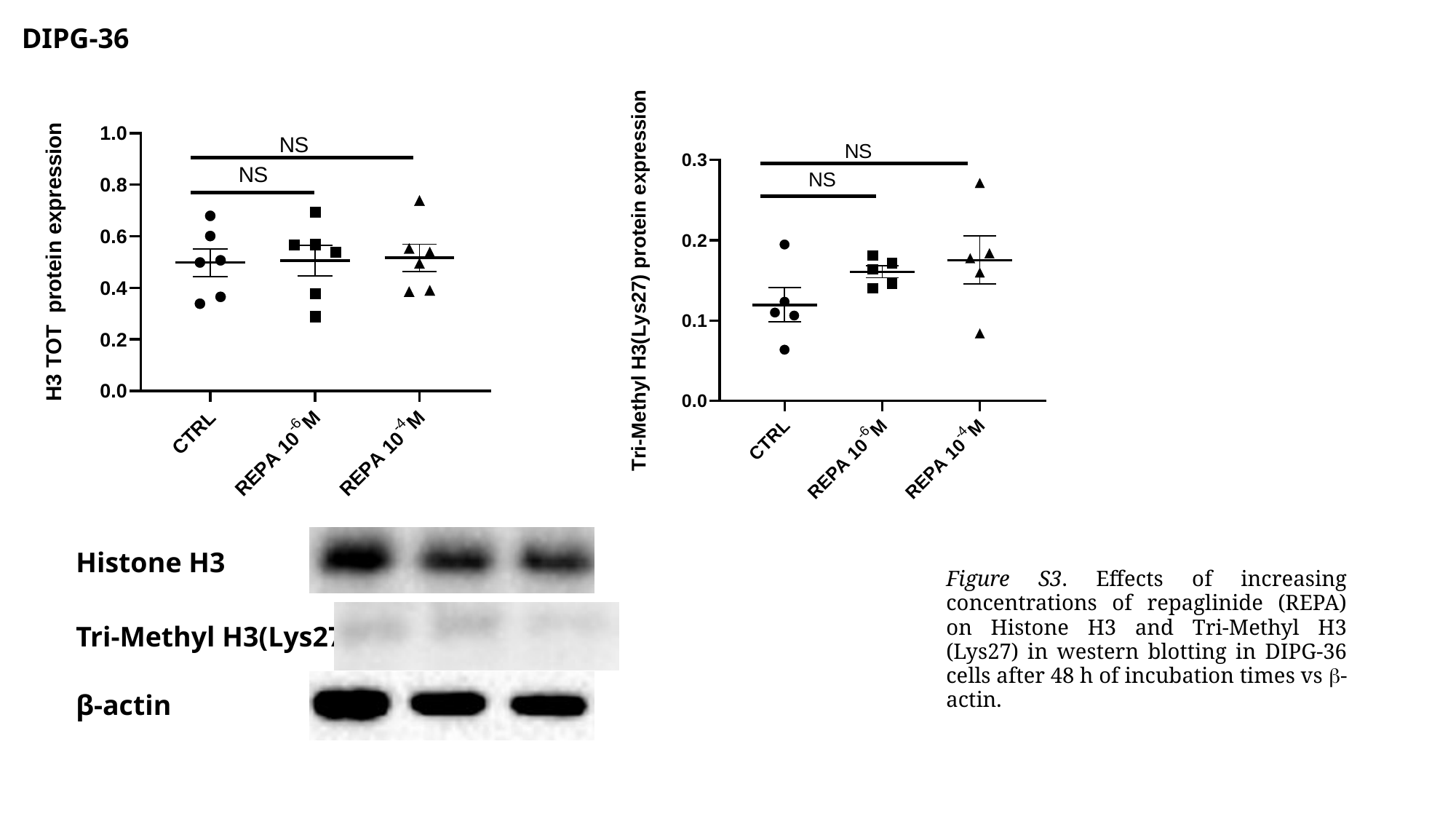

DIPG-36
Histone H3
Tri-Methyl H3(Lys27)
β-actin
Figure S3. Effects of increasing concentrations of repaglinide (REPA) on Histone H3 and Tri-Methyl H3 (Lys27) in western blotting in DIPG-36 cells after 48 h of incubation times vs b- actin.

## Slide 5
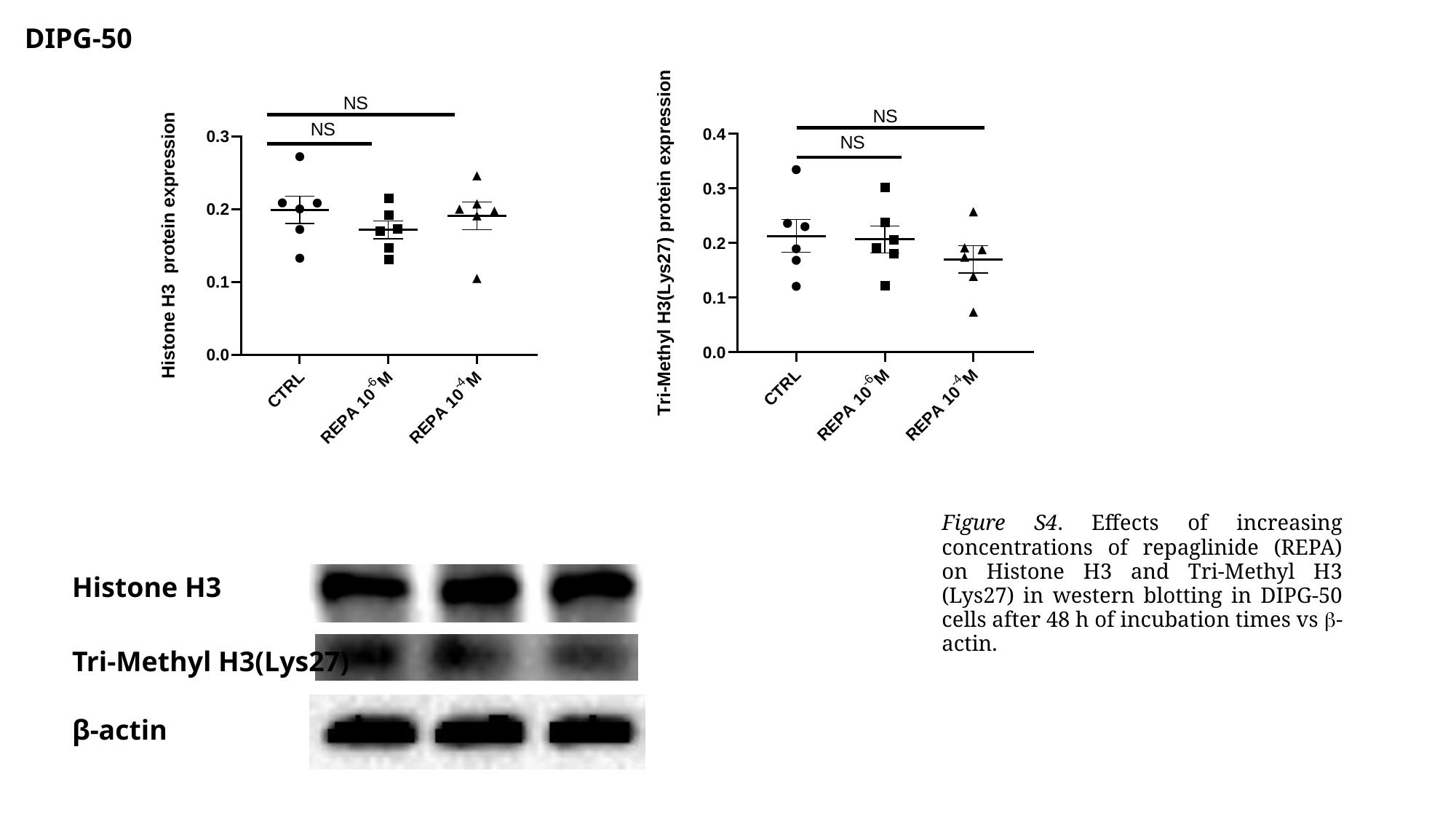

DIPG-50
Figure S4. Effects of increasing concentrations of repaglinide (REPA) on Histone H3 and Tri-Methyl H3 (Lys27) in western blotting in DIPG-50 cells after 48 h of incubation times vs b- actin.
Histone H3
Tri-Methyl H3(Lys27)
β-actin

## Slide 6
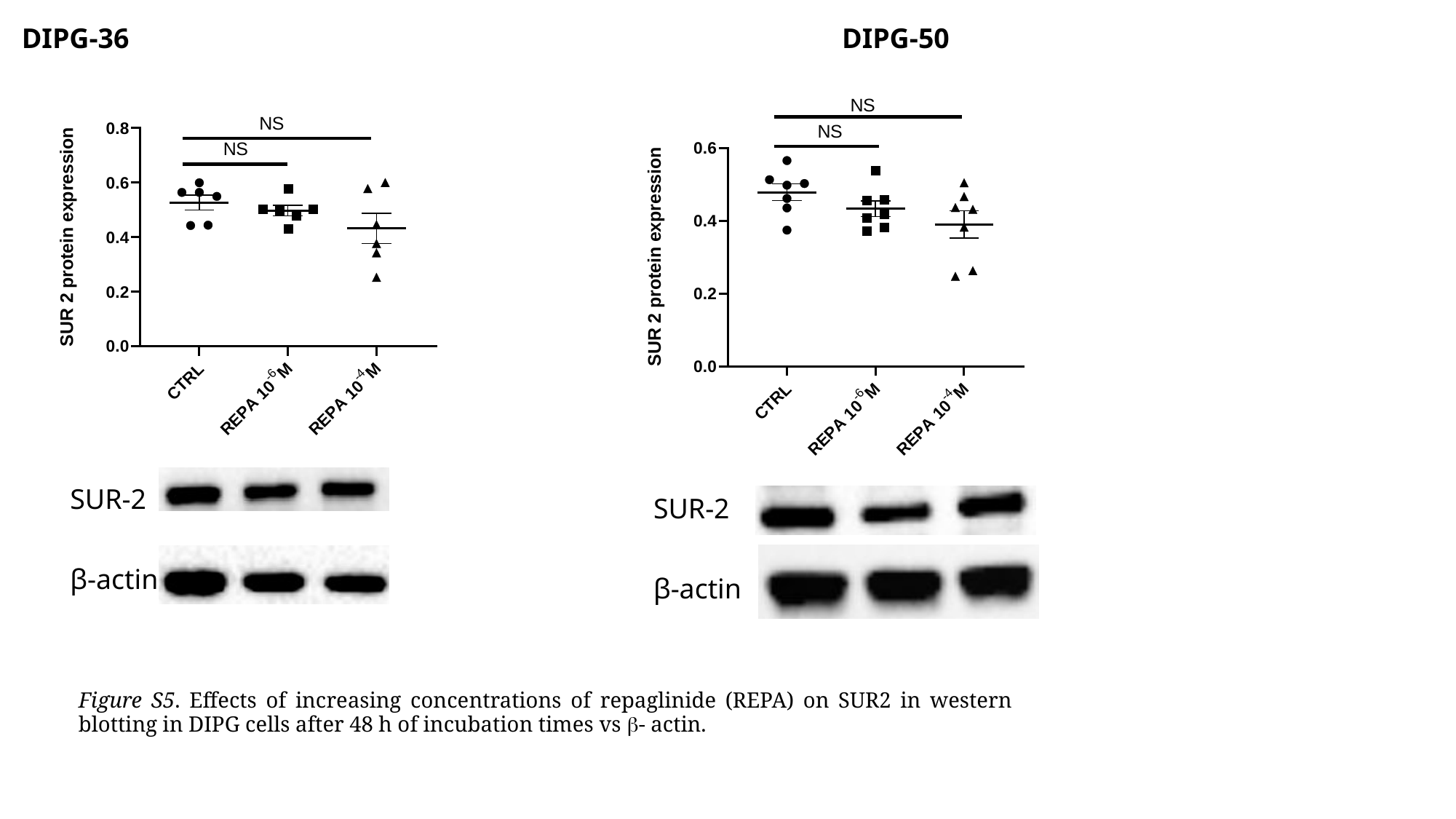

DIPG-36
DIPG-50
SUR-2
β-actin
SUR-2
β-actin
Figure S5. Effects of increasing concentrations of repaglinide (REPA) on SUR2 in western blotting in DIPG cells after 48 h of incubation times vs b- actin.
