# Supplementary material for: KATP Channel Inhibitors Reduce Cell Proliferation Through Upregulation of H3K27ac in Diffuse Intrinsic Pontine Glioma: A Functional Expression Investigation
Source: Cancers (Basel). 2025 Jan 22;17(3):358. doi: 10.3390/cancers17030358 (PMC11816144; doi:10.3390/cancers17030358)
Supplement: Supplementary file 1 [file cancers-17-00358-s001.zip › Supplementary File 3. (Western blot experiments).pdf]

Gel -1

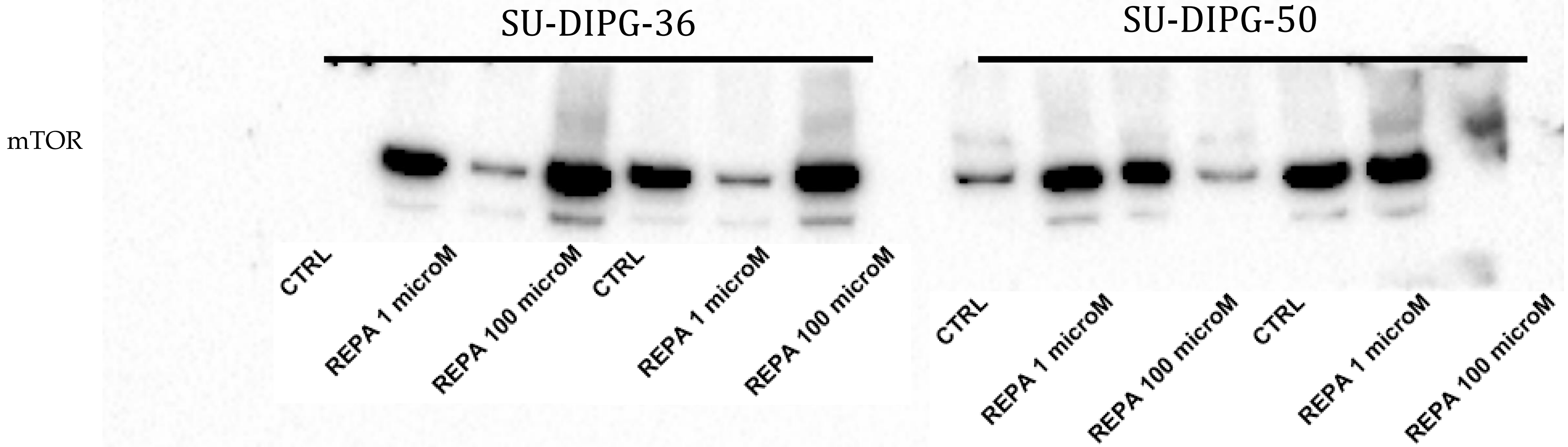

SU-DIPG-36
SU-DIPG-50
mTOR
CTRL
REPA 1 microM
REPA 100 microM
CTRL
REPA 1 microM
REPA 100 microM
CTRL
REPA 1 microM
REPA 100 microM
CTRL
REPA 1 microM
REPA 100 microM

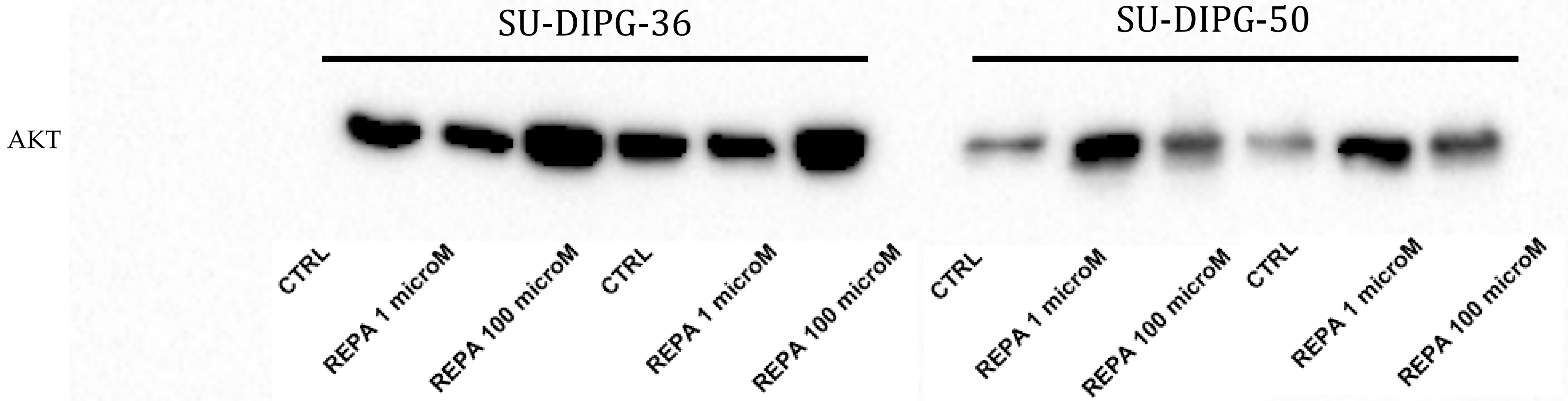
SU-DIPG-36
SU-DIPG-50
AKT
CTRL
REPA 1 microM
REPA 100 microM
CTRL
REPA 1 microM
REPA 100 microM
CTRL
REPA 1 microM
REPA 100 microM
CTRL
REPA 1 microM
REPA 100 microM

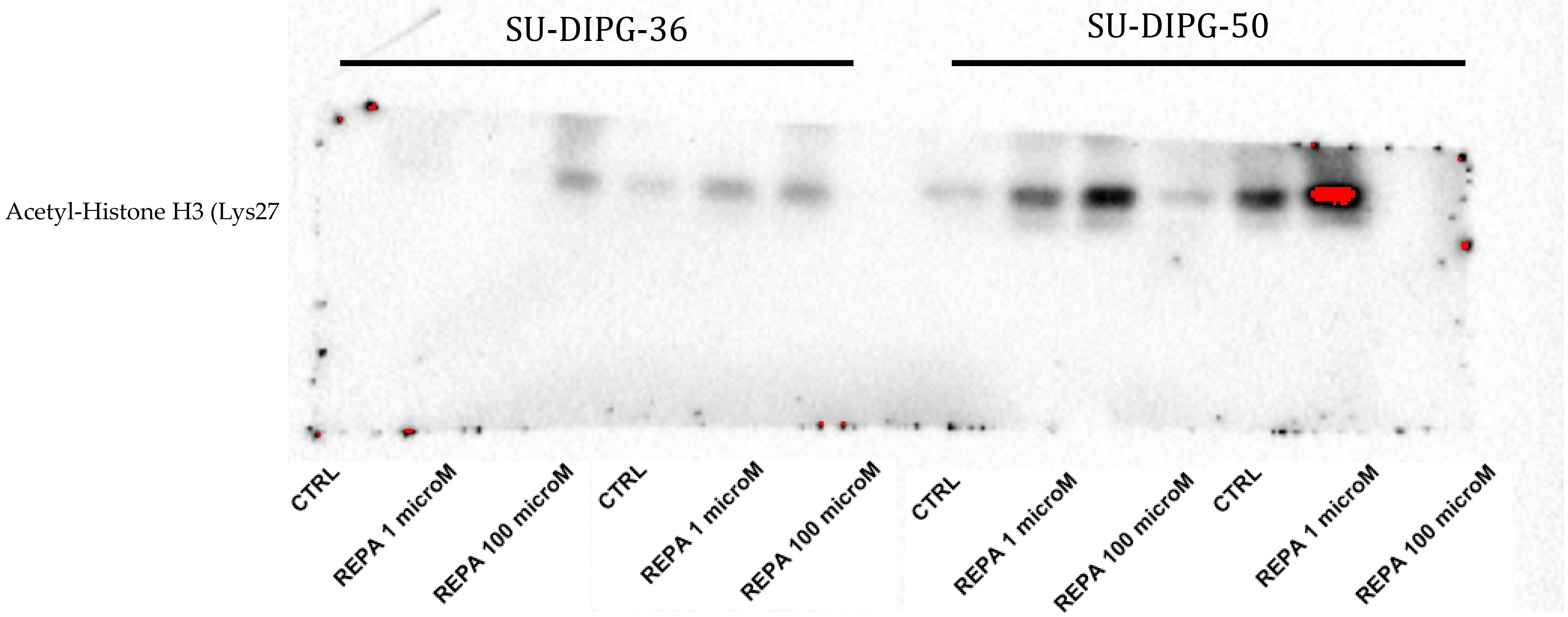
SU-DIPG-36
SU-DIPG-50
Acetyl-Histone H3 (Lys27
CTRL
REPA 1 microM
REPA 100 microM
CTRL
REPA 1 microM
REPA 100 microM
CTRL
REPA 1 microM
REPA 100 microM
CTRL
REPA 1 microM
REPA 100 microM

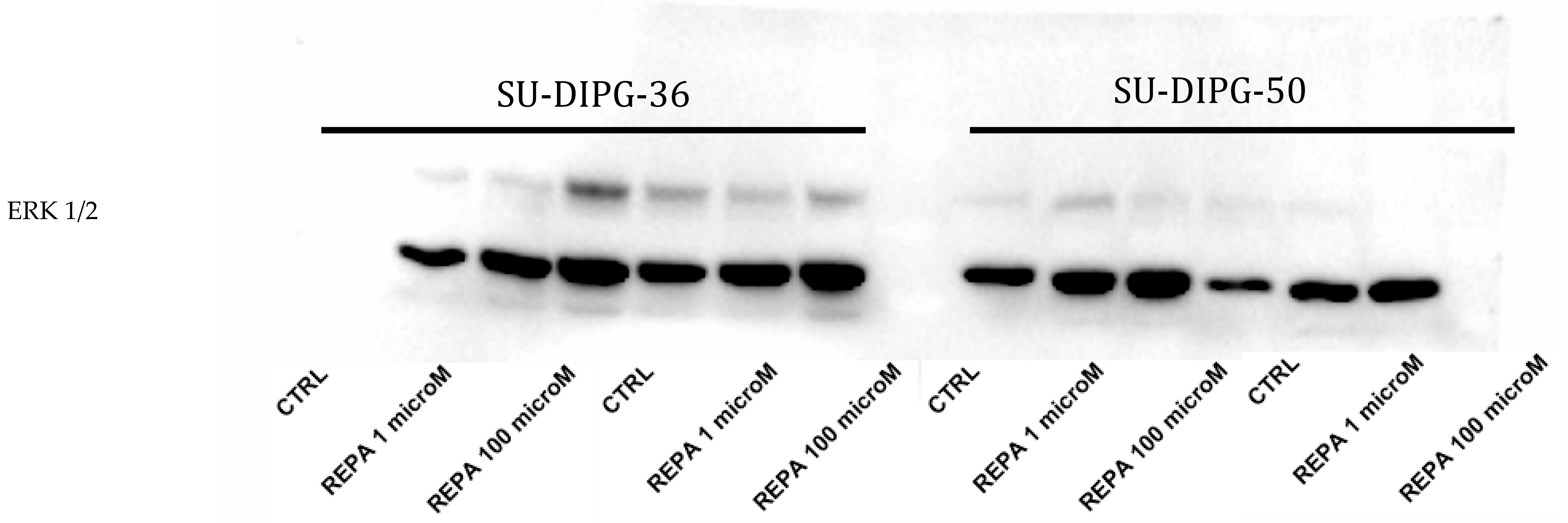
SU-DIPG-36
SU-DIPG-50
ERK 1/2
CTRL
REPA 1 microM
REPA 100 microM
CTRL
REPA 1 microM
REPA 100 microM
CTRL
REPA 1 microM
REPA 100 microM
CTRL
REPA 1 microM
REPA 100 microM

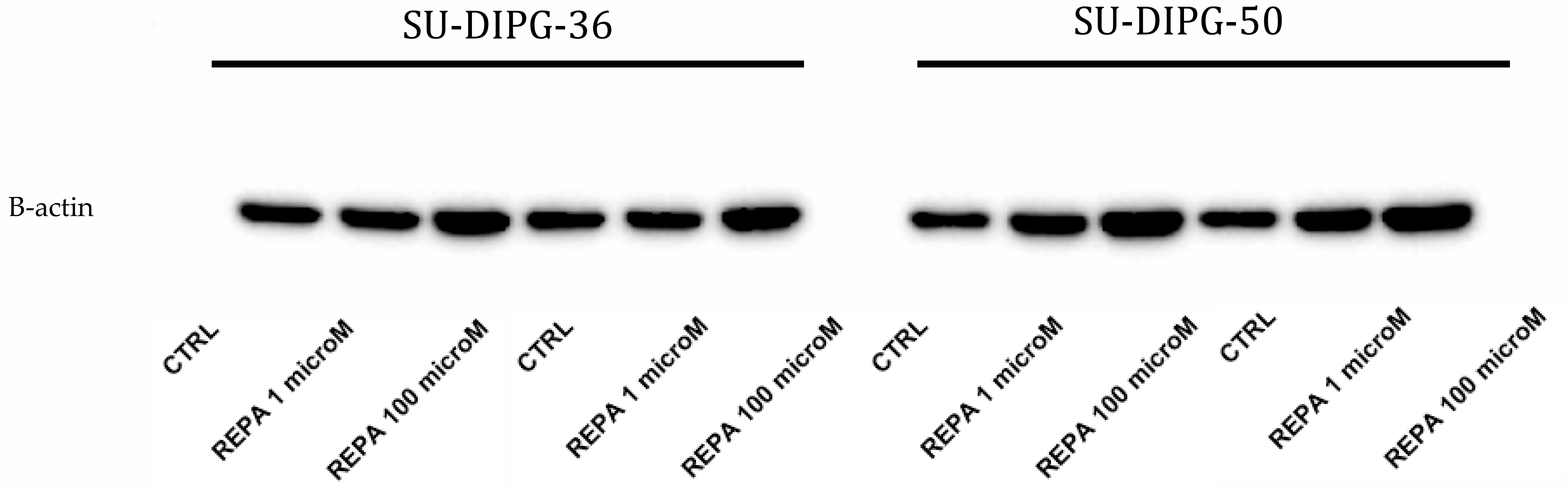
SU-DIPG-36
SU-DIPG-50
B-actin
CTRL
REPA 1 microM
REPA 100 microM
CTRL
REPA 1 microM
REPA 100 microM
CTRL
REPA 1 microM
REPA 100 microM
CTRL
REPA 1 microM
REPA 100 microM

Gel -2

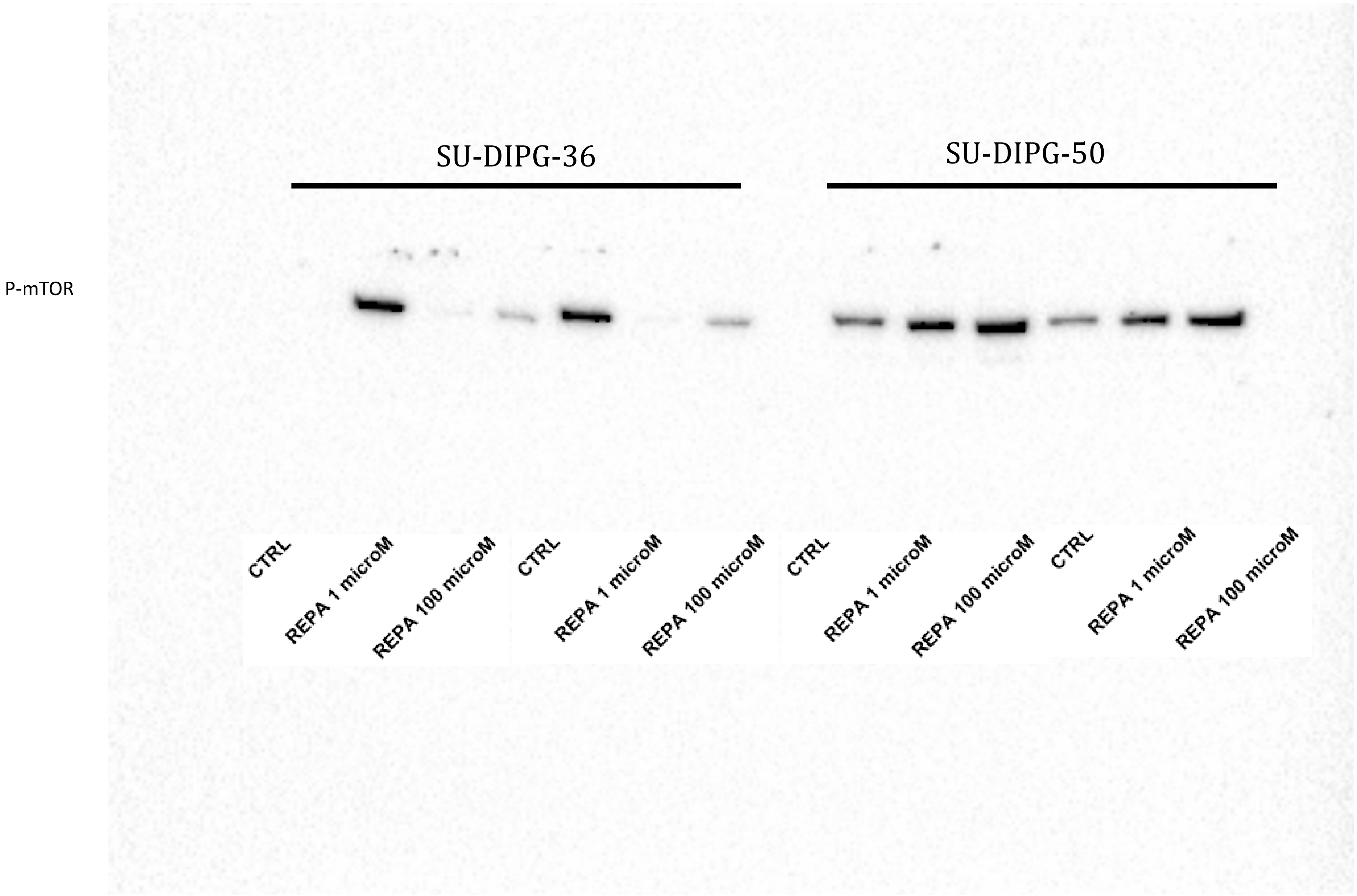
SU-DIPG-36
SU-DIPG-50
P-mTOR
CTRL
REPA 1 microM
REPA 100 microM
CTRL
REPA 1 microM
REPA 100 microM
CTRL
REPA 1 microM
REPA 100 microM
CTRL
REPA 1 microM
REPA 100 microM

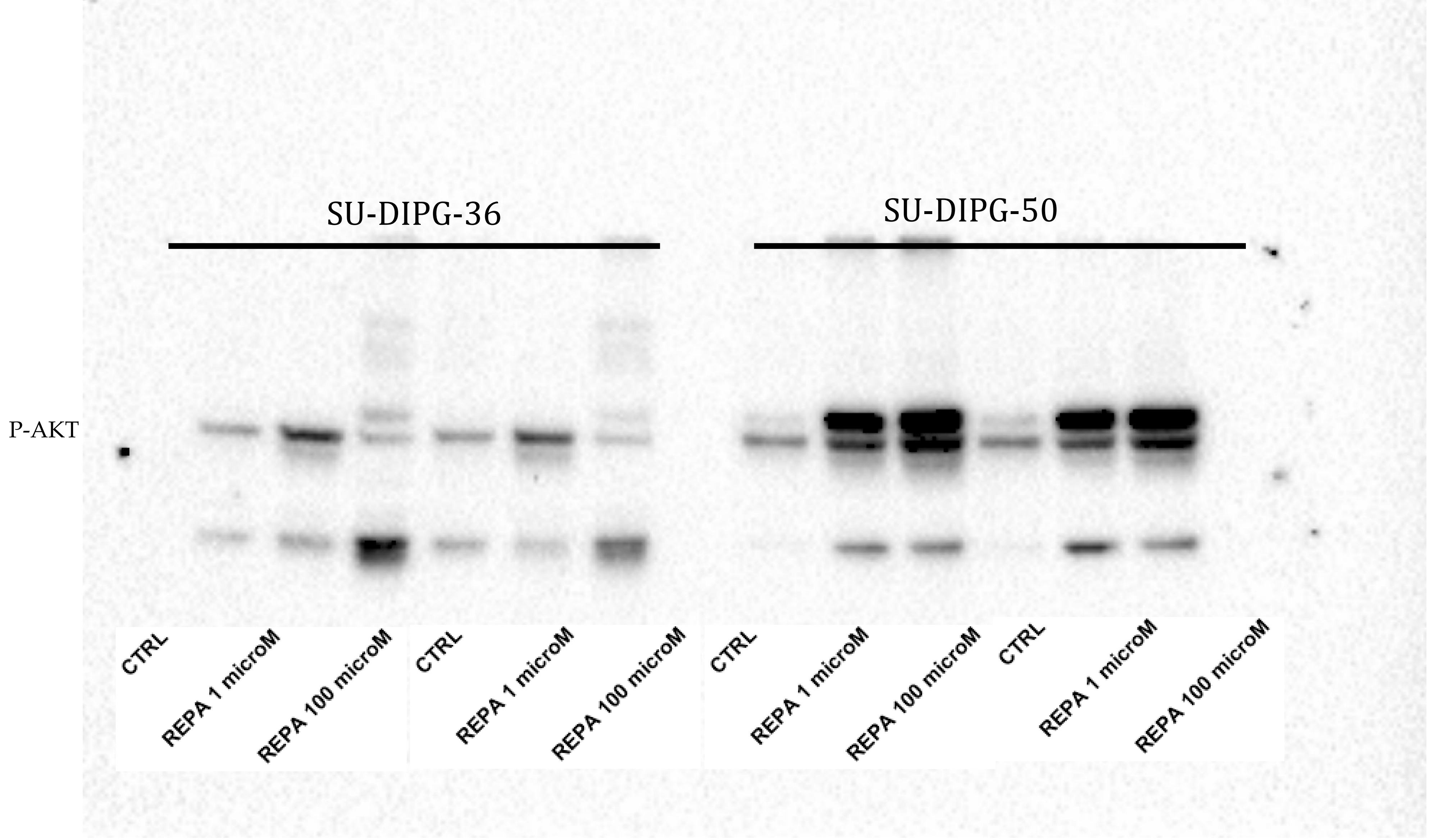
SU-DIPG-36
SU-DIPG-50
P-AKT
CTRL
REPA 1 microM
REPA 100 microM
CTRL
REPA 1 microM
REPA 100 microM
CTRL
REPA 1 microM
REPA 100 microM
CTRL
REPA 1 microM
REPA 100 microM

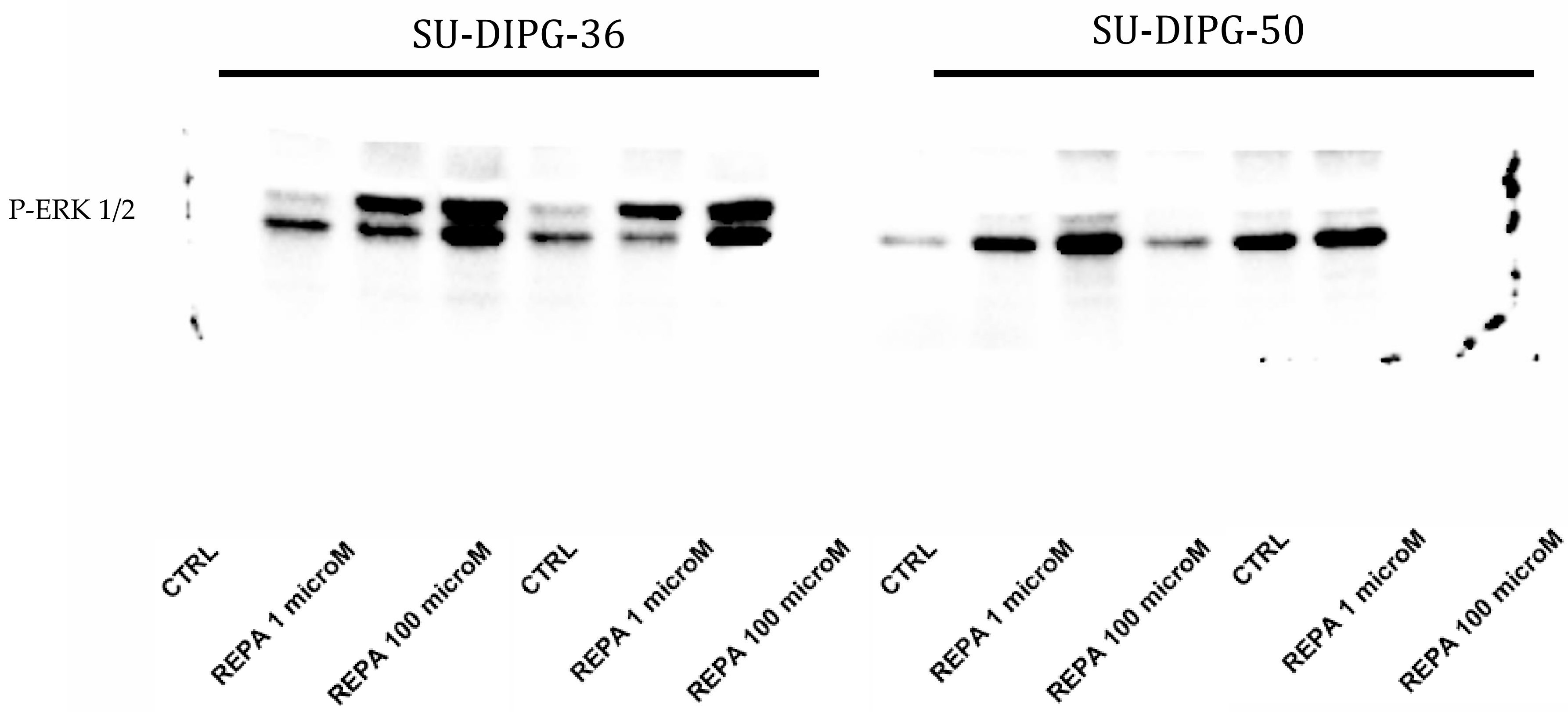

SU-DIPG-36
SU-DIPG-50
P-ERK 1/2
CTRL
REPA 1 microM
REPA 100 microM
CTRL
REPA 1 microM
REPA 100 microM
CTRL
REPA 1 microM
REPA 100 microM
CTRL
REPA 1 microM
REPA 100 microM

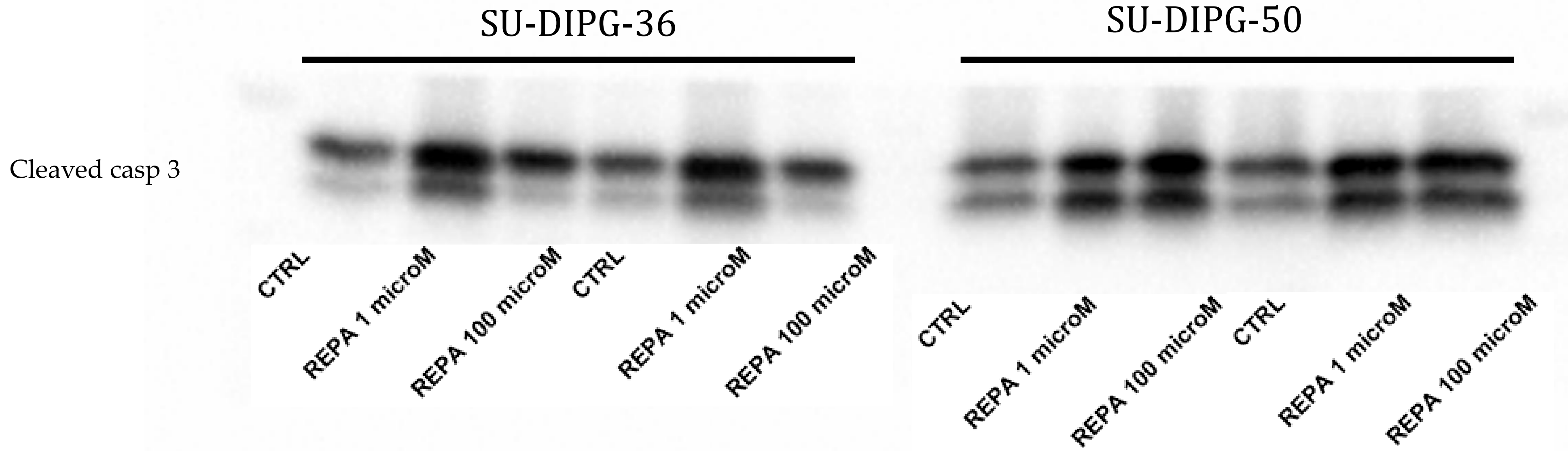
SU-DIPG-36
SU-DIPG-50
Cleaved casp 3
CTRL
REPA 1 microM
REPA 100 microM
CTRL
REPA 1 microM
REPA 100 microM
CTRL
REPA 1 microM
REPA 100 microM
CTRL
REPA 1 microM
REPA 100 microM

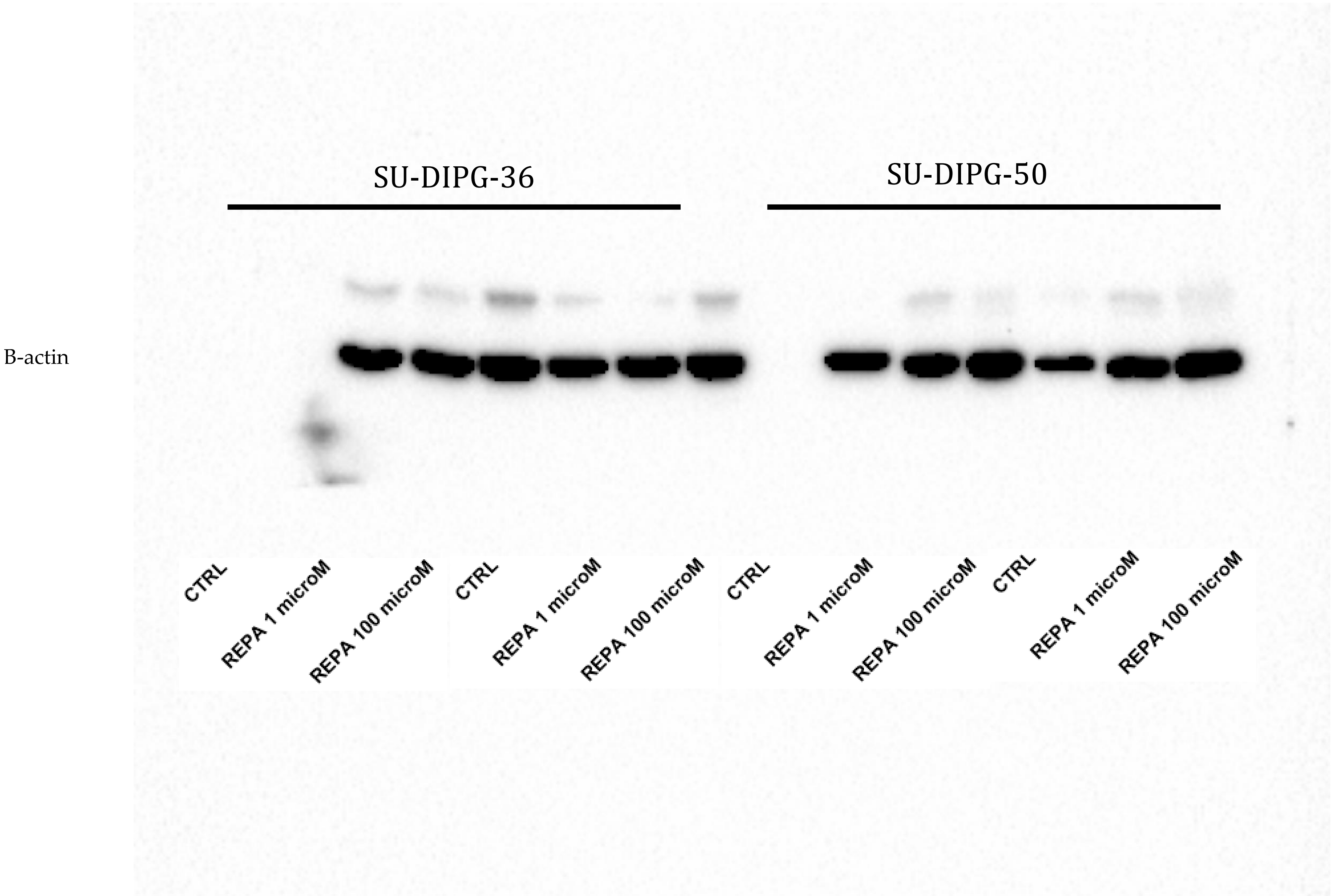
SU-DIPG-36
SU-DIPG-50
B-actin
CTRL
REPA 1 microM
REPA 100 microM
CTRL
REPA 1 microM
REPA 100 microM
CTRL
REPA 1 microM
REPA 100 microM
CTRL
REPA 1 microM
REPA 100 microM

Gel -3

SU-DIPG-36

SU-DIPG-50

P-erk 1/2

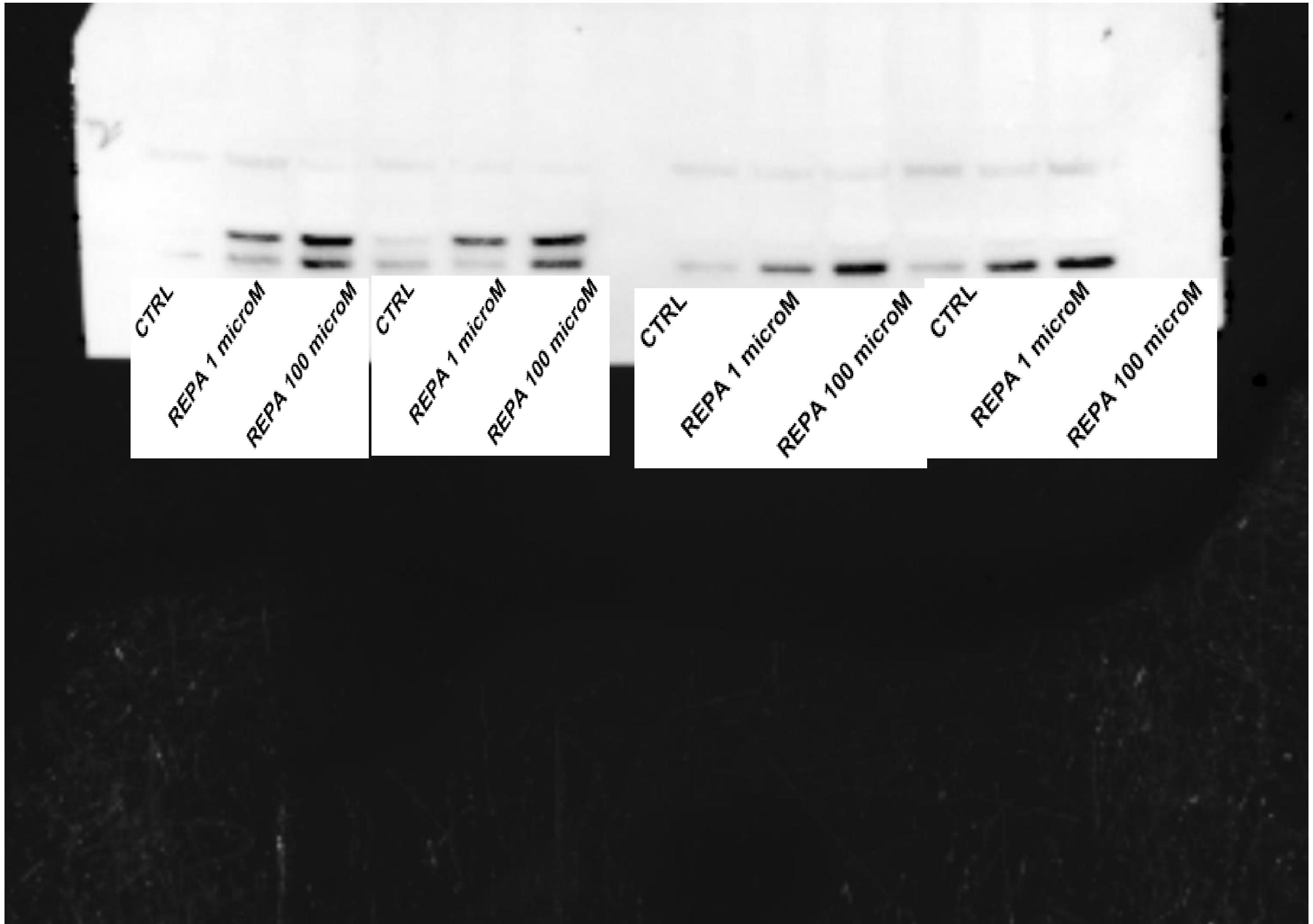

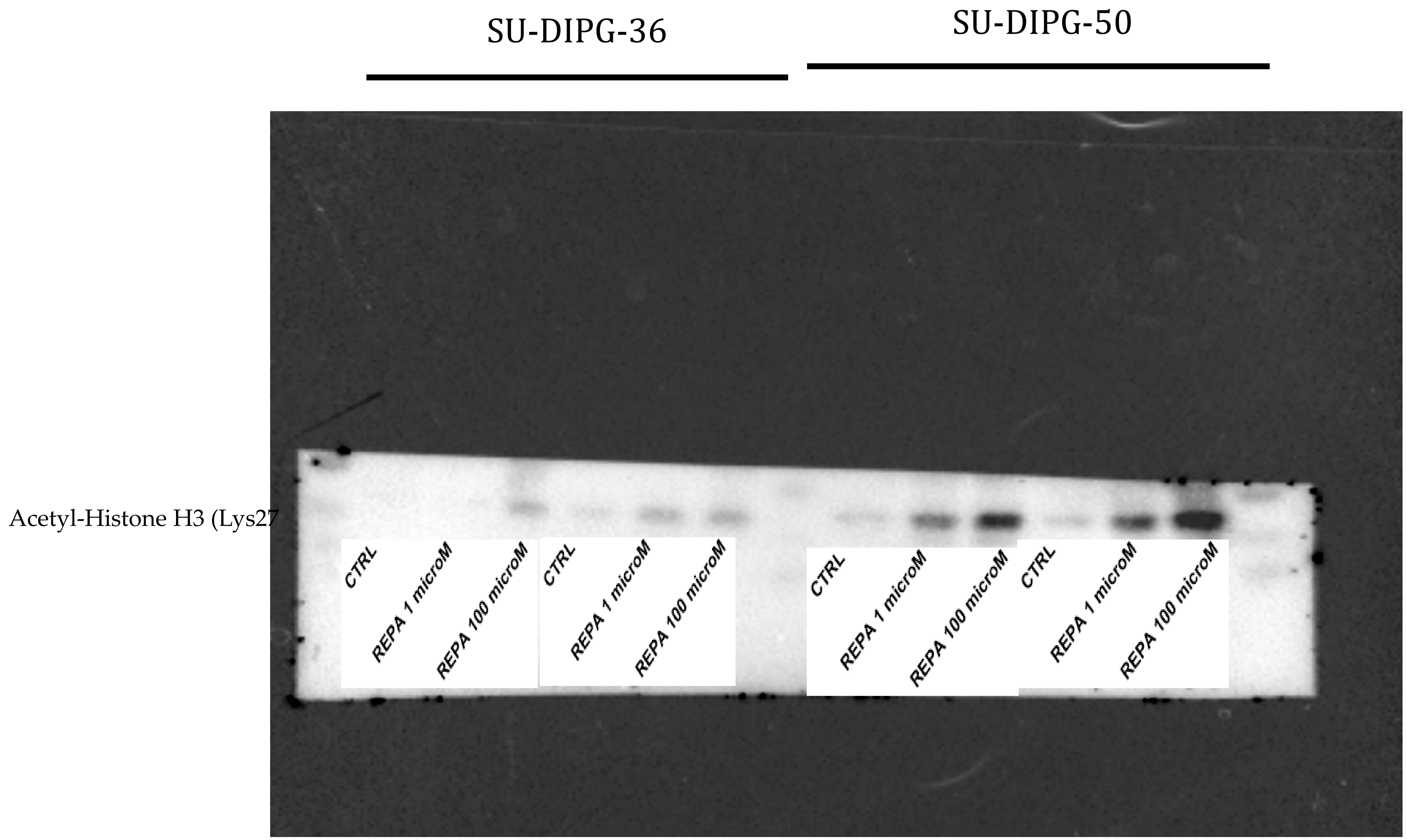
SU-DIPG-36
SU-DIPG-50
Acetyl-Histone H3 (Lys27
CTRL
REPA 1 microM
REPA 100 microM
CTRL
REPA 1 microM
REPA 100 microM
CTRL
REPA 1 microM
REPA 100 microM
CTRL
REPA 1 microM
REPA 100 microM

B-actin

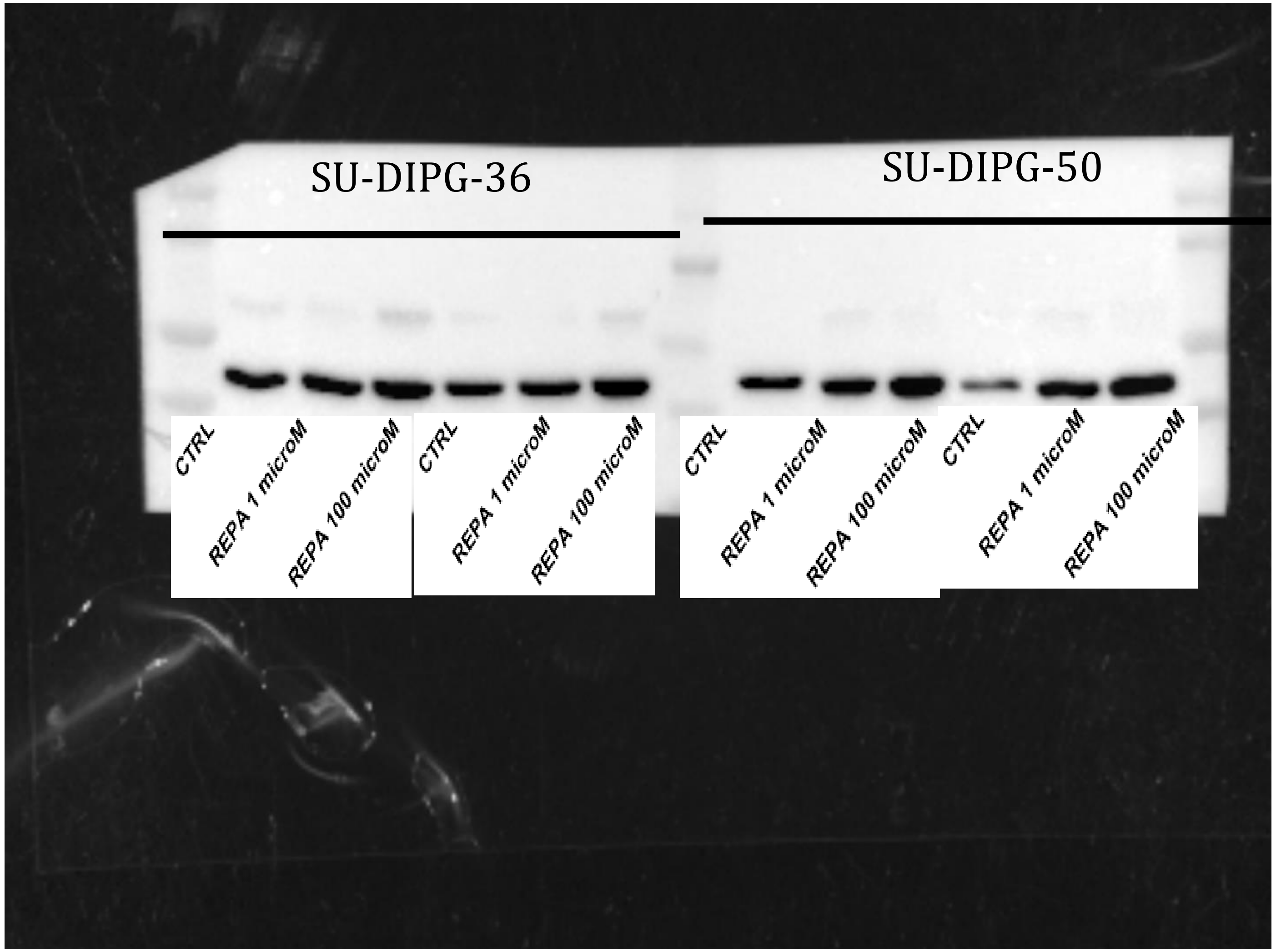

SU-DIPG-36
SU-DIPG-50
CTRL
REPA 1 microM
REPA 100 microM
CTRL
REPA 1 microM
REPA 100 microM
CTRL
REPA 1 microM
REPA 100 microM
CTRL
REPA 1 microM
REPA 100 microM

Gel -4

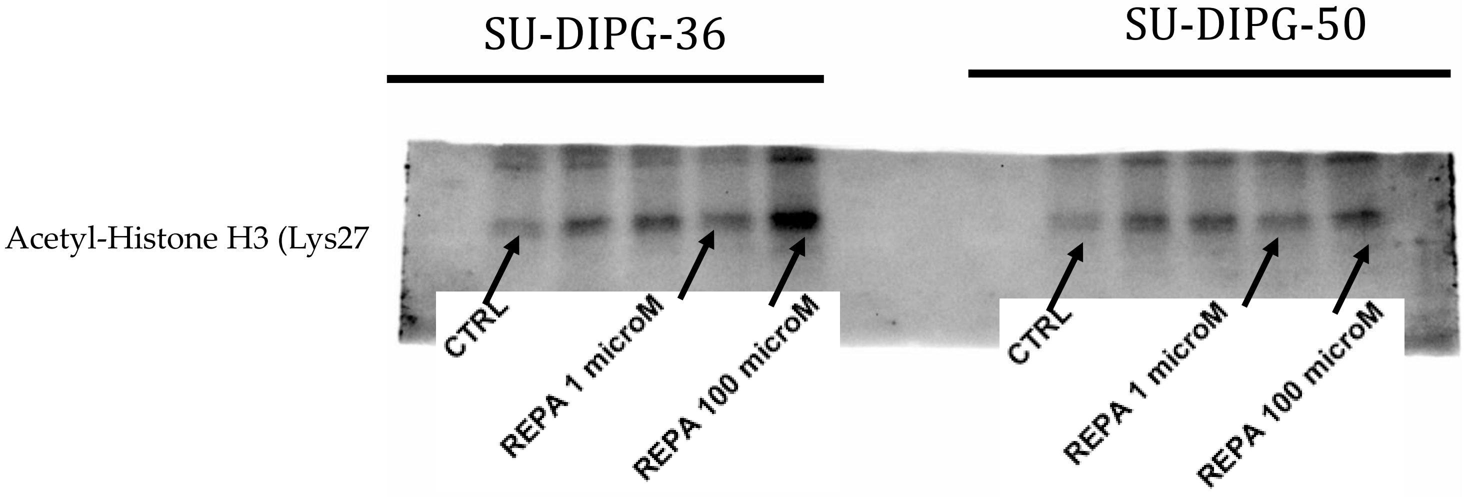
SU-DIPG-36
SU-DIPG-50
Acetyl-Histone H3 (Lys27
CTRL
REPA 1 microM
REPA 100 microM
CTRL
REPA 1 microM
REPA 100 microM

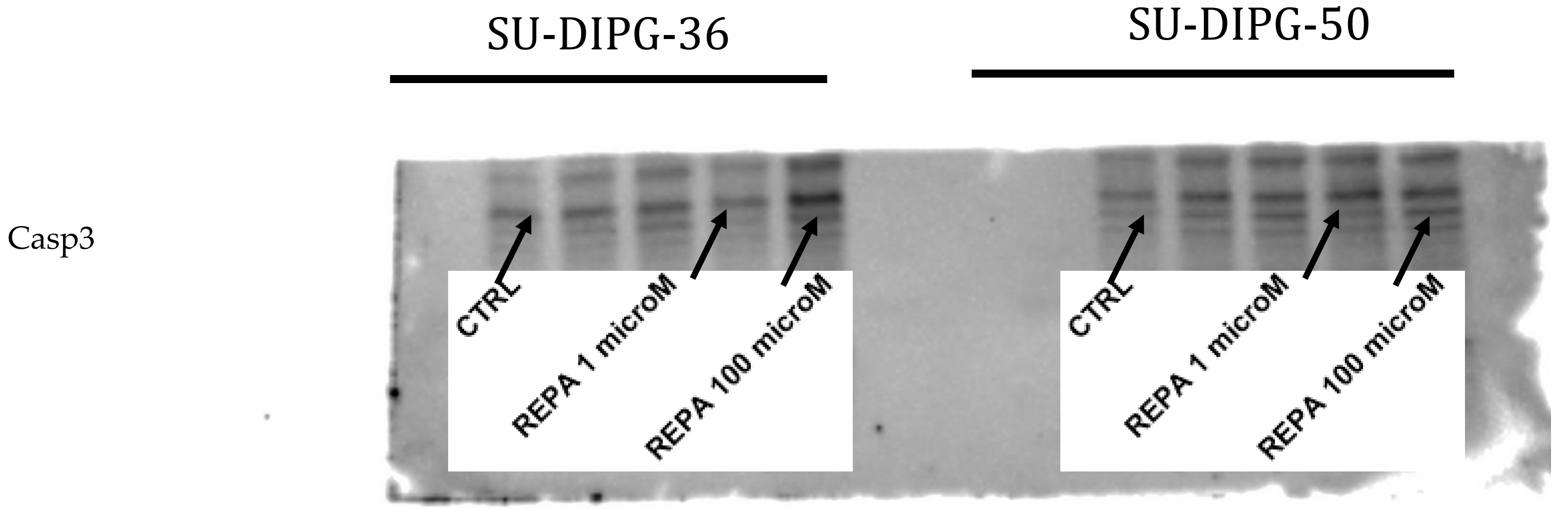

SU-DIPG-36
SU-DIPG-50
Casp3
CTRL
REPA 1 microM
REPA 100 microM
CTRL
REPA 1 microM
REPA 100 microM

Casp3

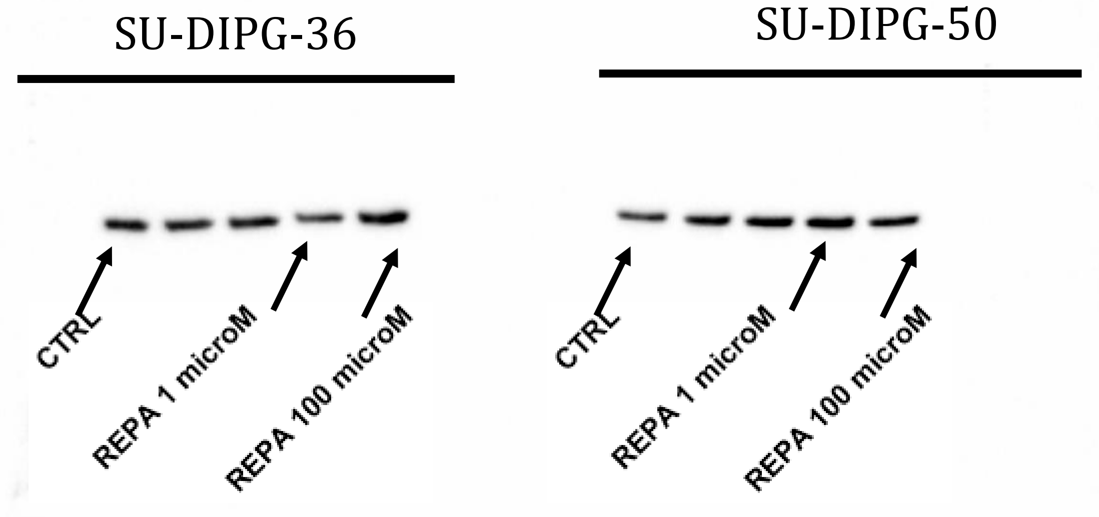
SU-DIPG-36
SU-DIPG-50
CTRL
REPA 1 microM
REPA 100 microM
CTRL
REPA 1 microM
REPA 100 microM

Gel -5

Acetyl-Histone H3 (Lys27

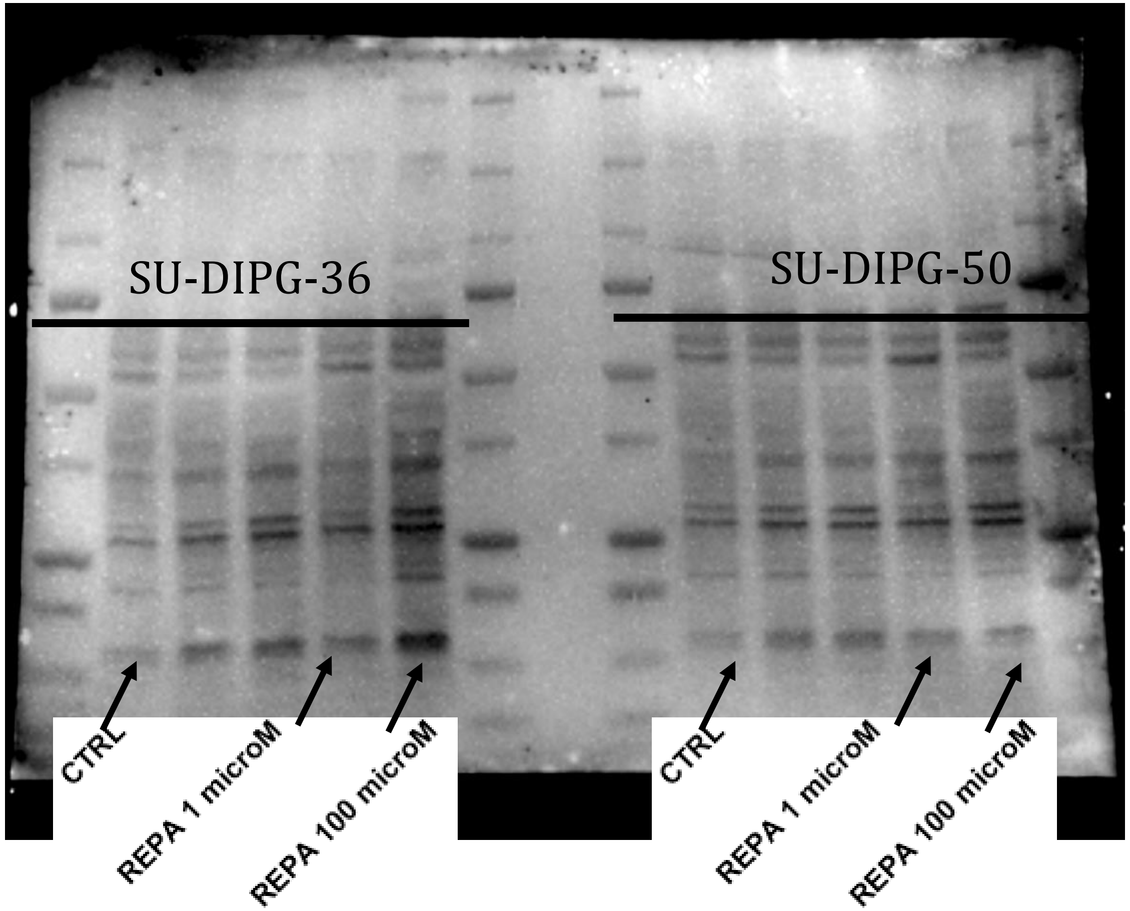

B-ACTIN

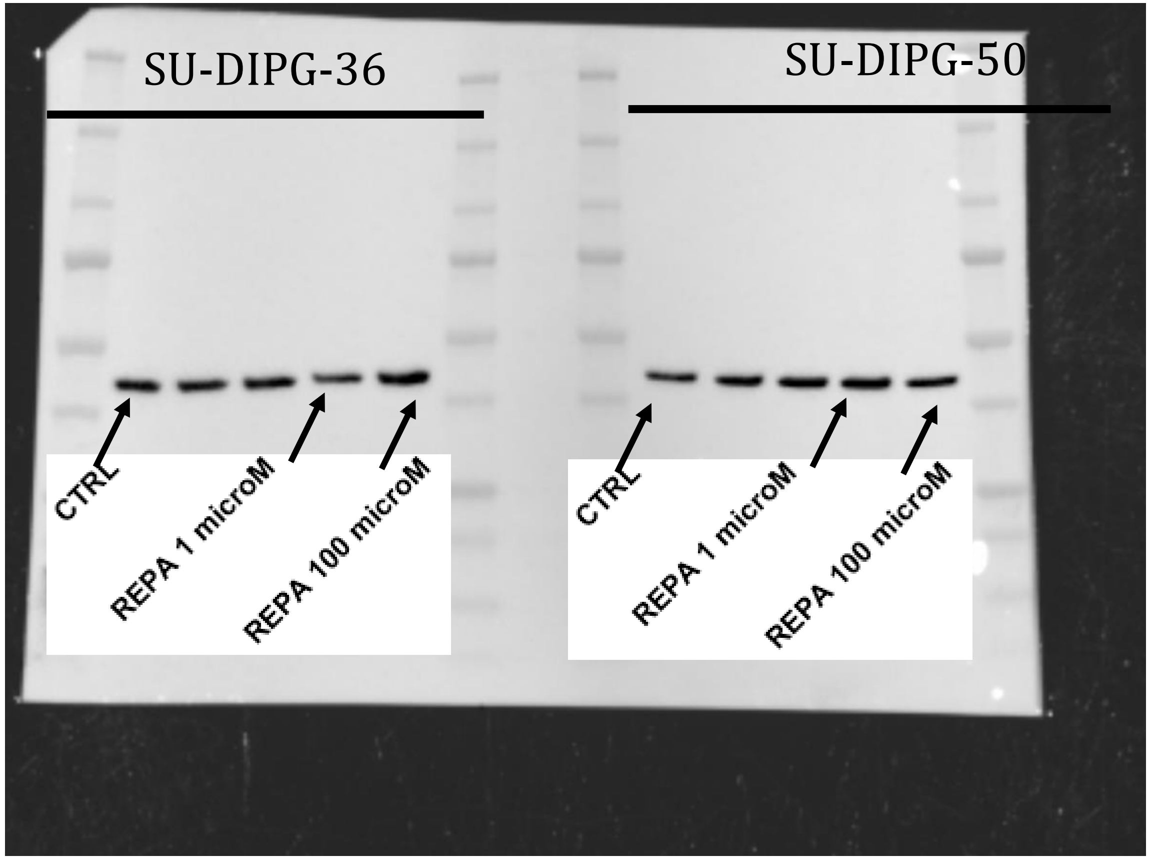
SU-DIPG-36
SU-DIPG-50
CTRL
REPA 1 microM
REPA 100 microM
CTRL
REPA 1 microM
REPA 100 microM

Gel -6

AKT

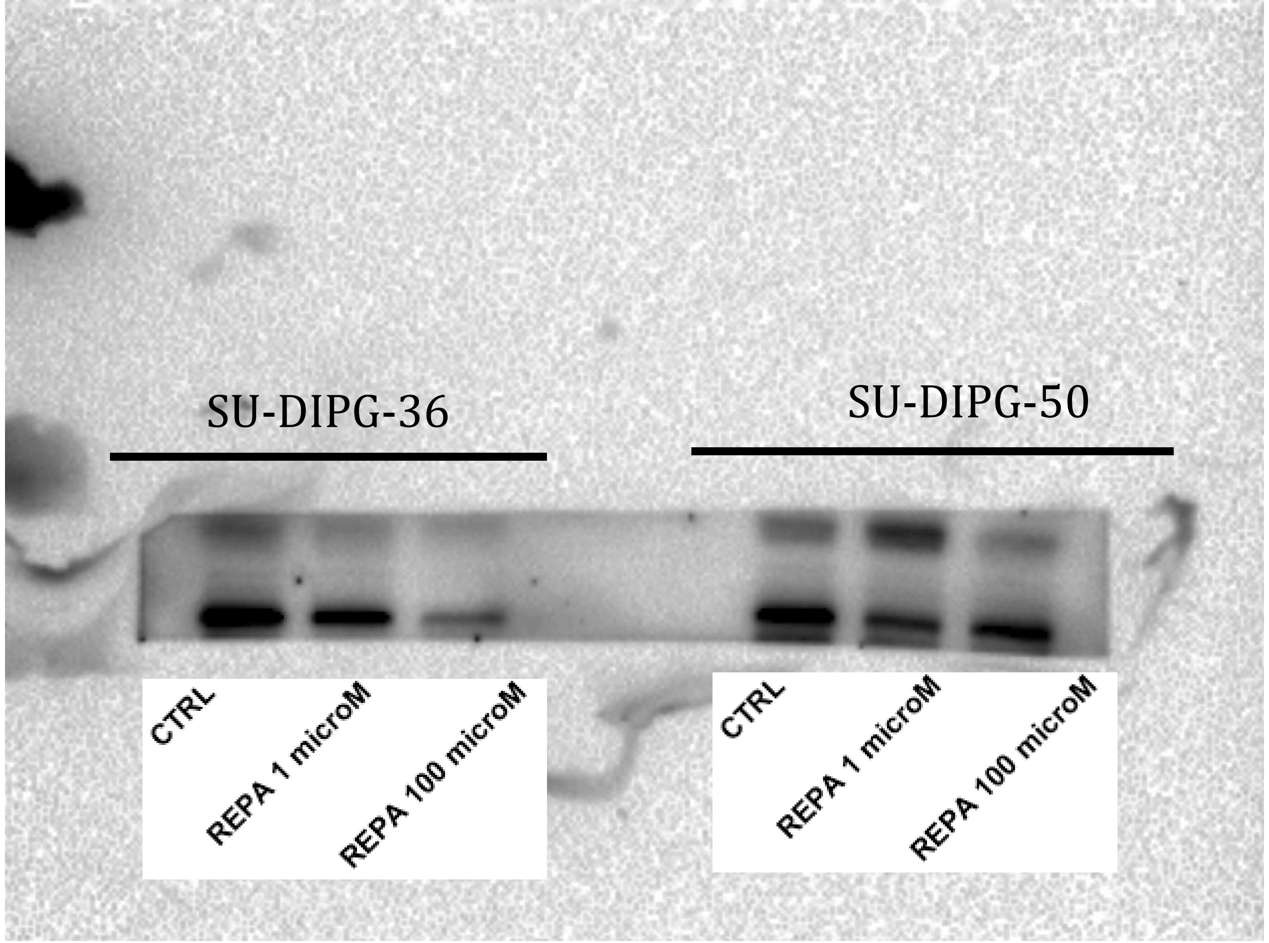
SU-DIPG-36
SU-DIPG-50
CTRL
REPA 1 microM
REPA 100 microM
CTRL
REPA 1 microM
REPA 100 microM

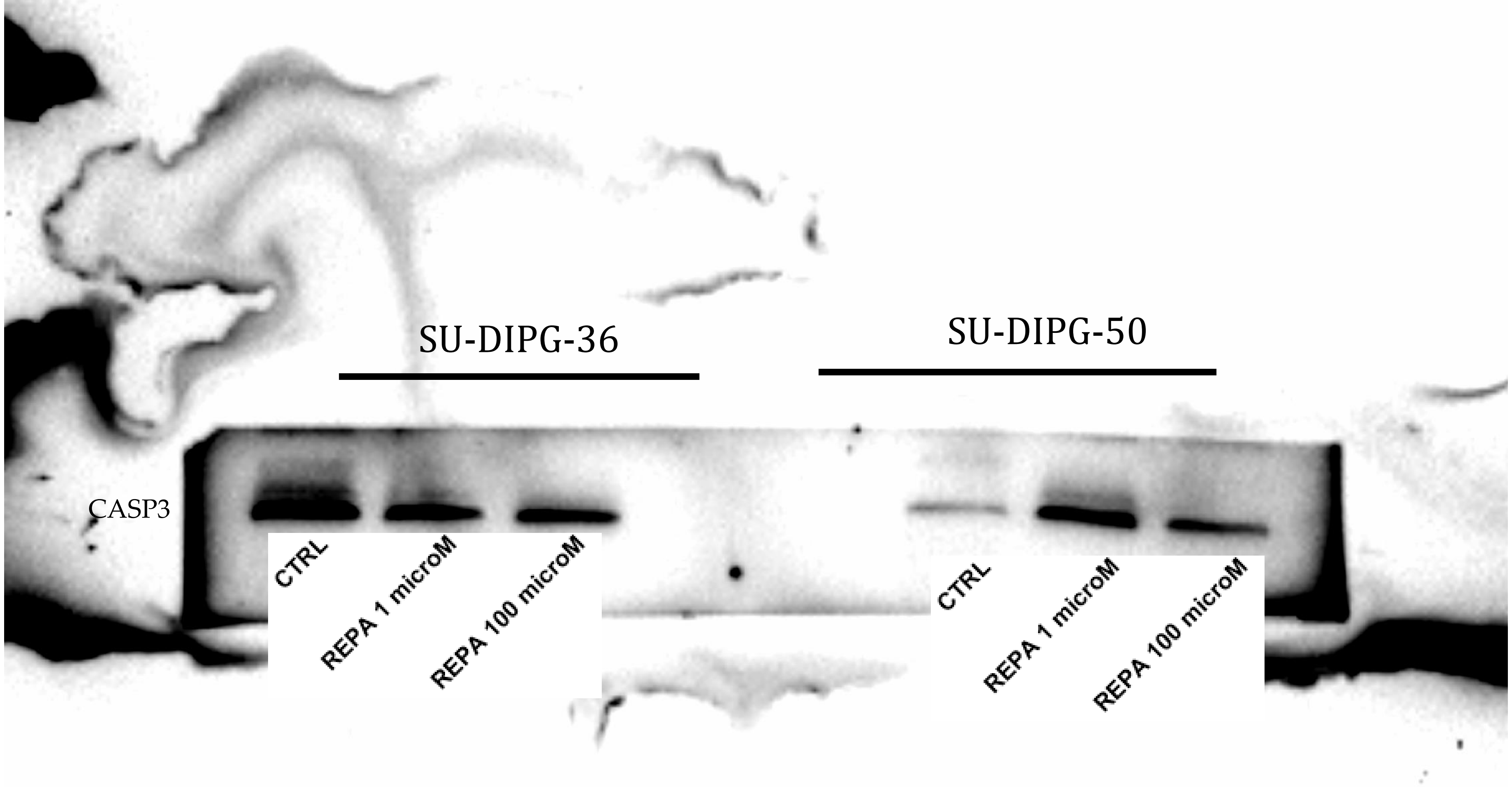
SU-DIPG-36
SU-DIPG-50
CASP3
CTRL
REPA 1 microM
REPA 100 microM
CTRL
REPA 1 microM
REPA 100 microM

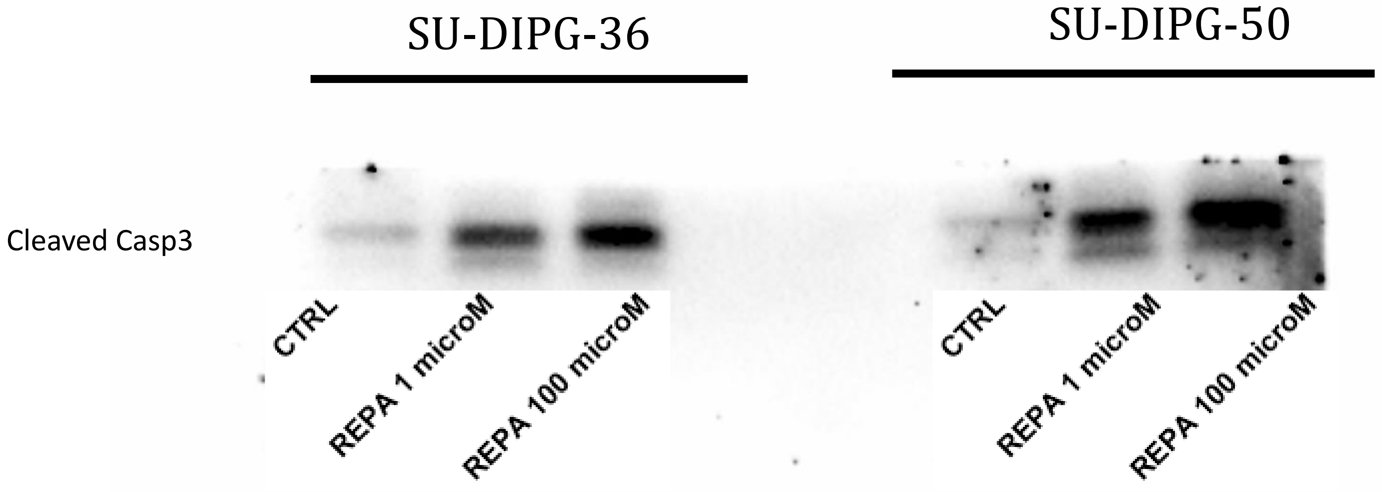

SU-DIPG-36
SU-DIPG-50
Cleaved Casp3
CTRL
REPA 1 microM
REPA 100 microM
CTRL
REPA 1 microM
REPA 100 microM

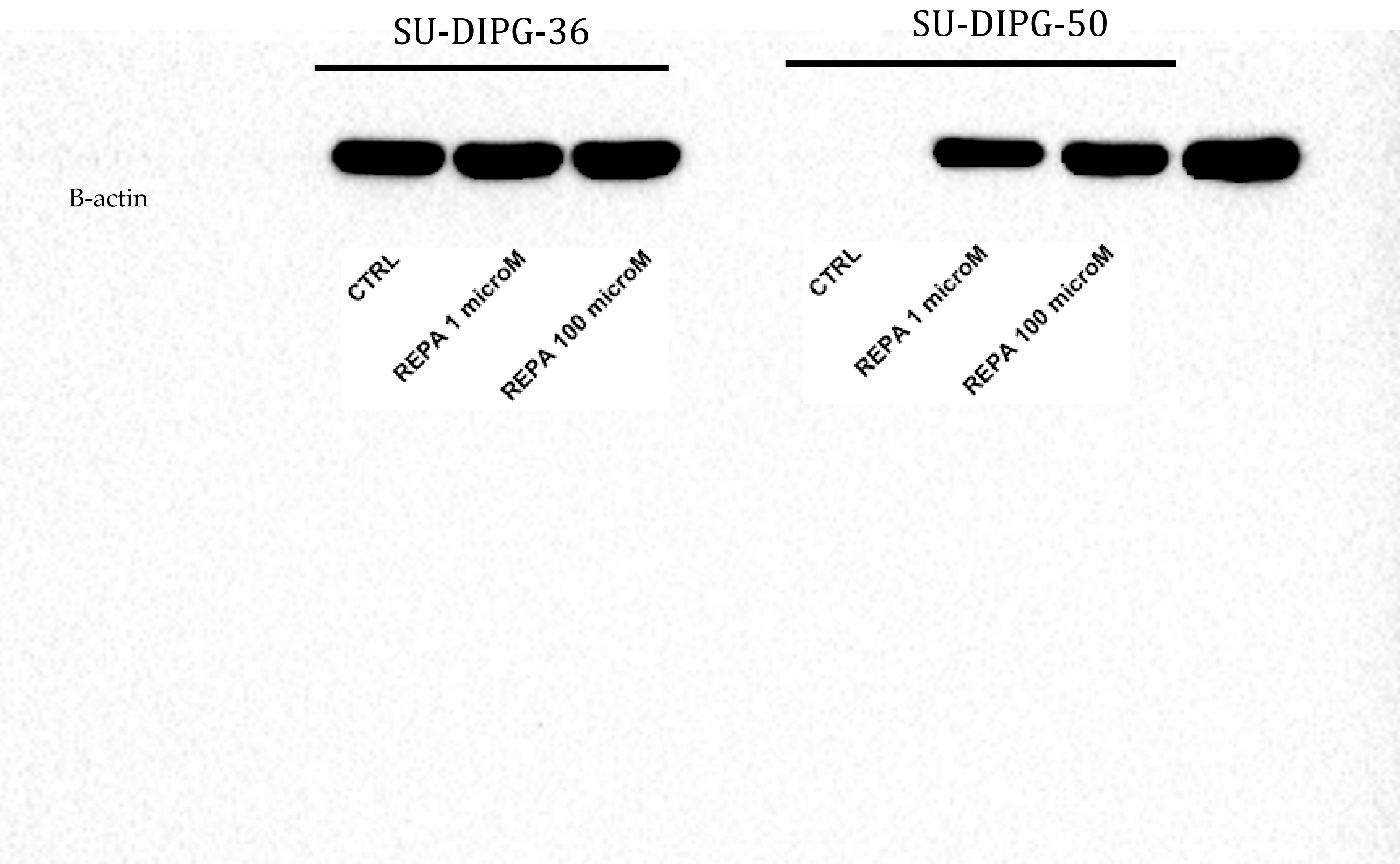
SU-DIPG-36
SU-DIPG-50
B-actin
CTRL
REPA 1 microM
REPA 100 microM
CTRL
REPA 1 microM
REPA 100 microM

Gel -7

P-mTOR

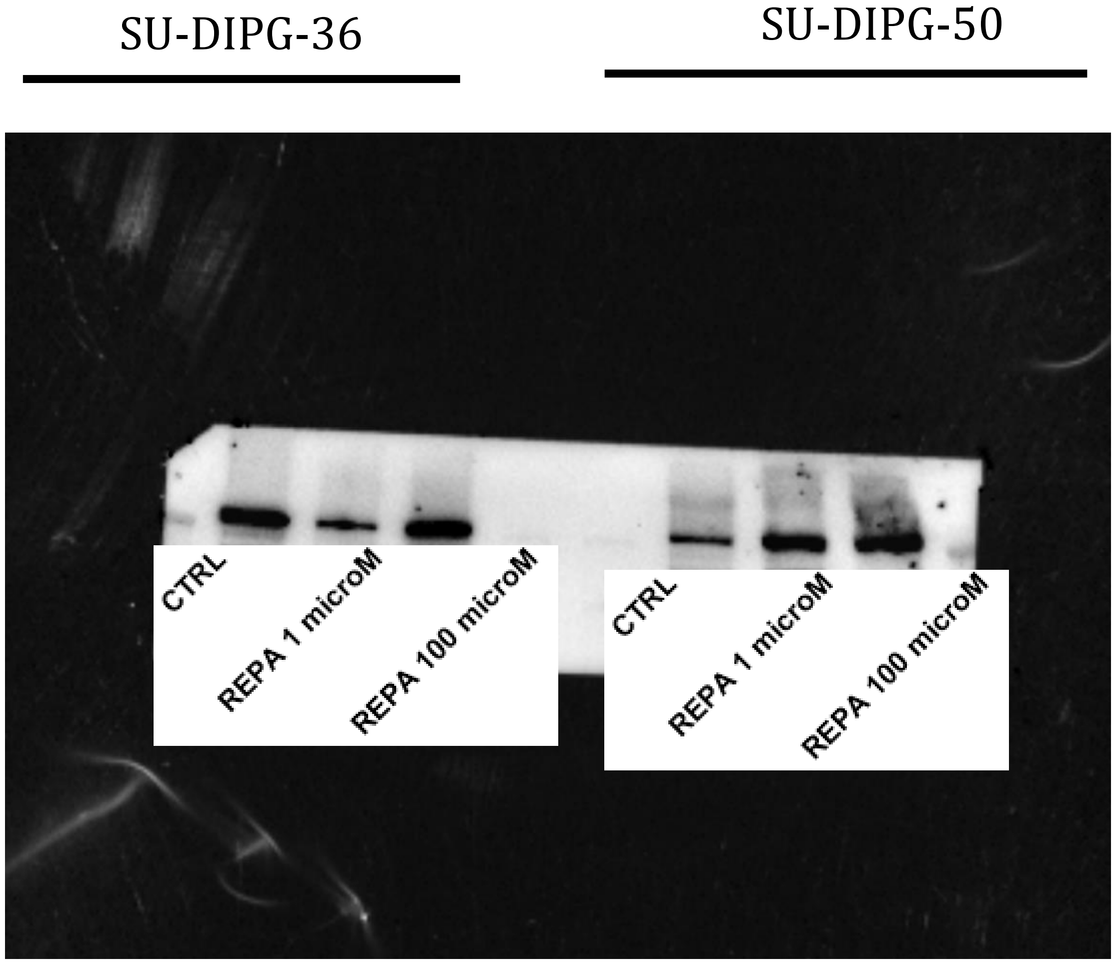
SU-DIPG-36
SU-DIPG-50
CTRL
REPA 1 microM
REPA 100 microM
CTRL
REPA 1 microM
REPA 100 microM

Cleaved Casp 3

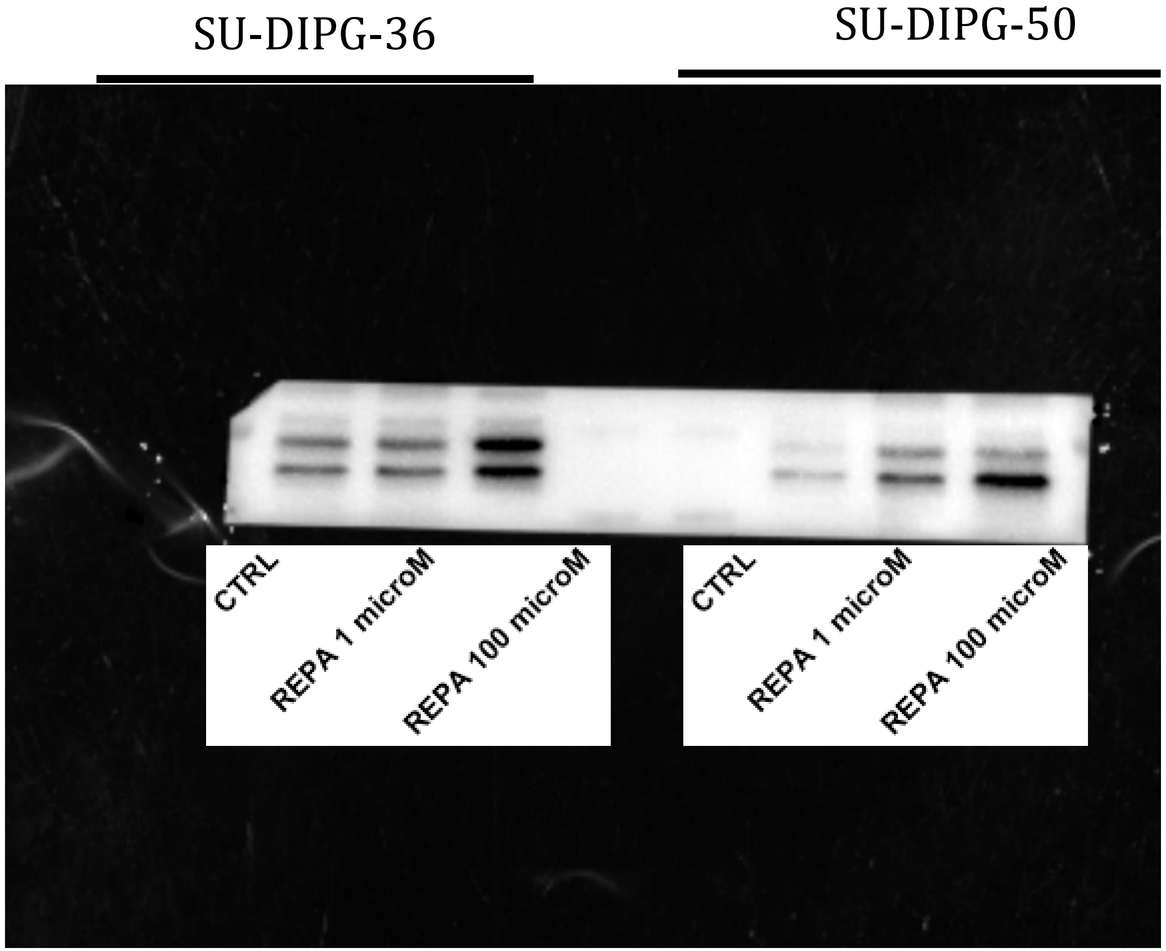

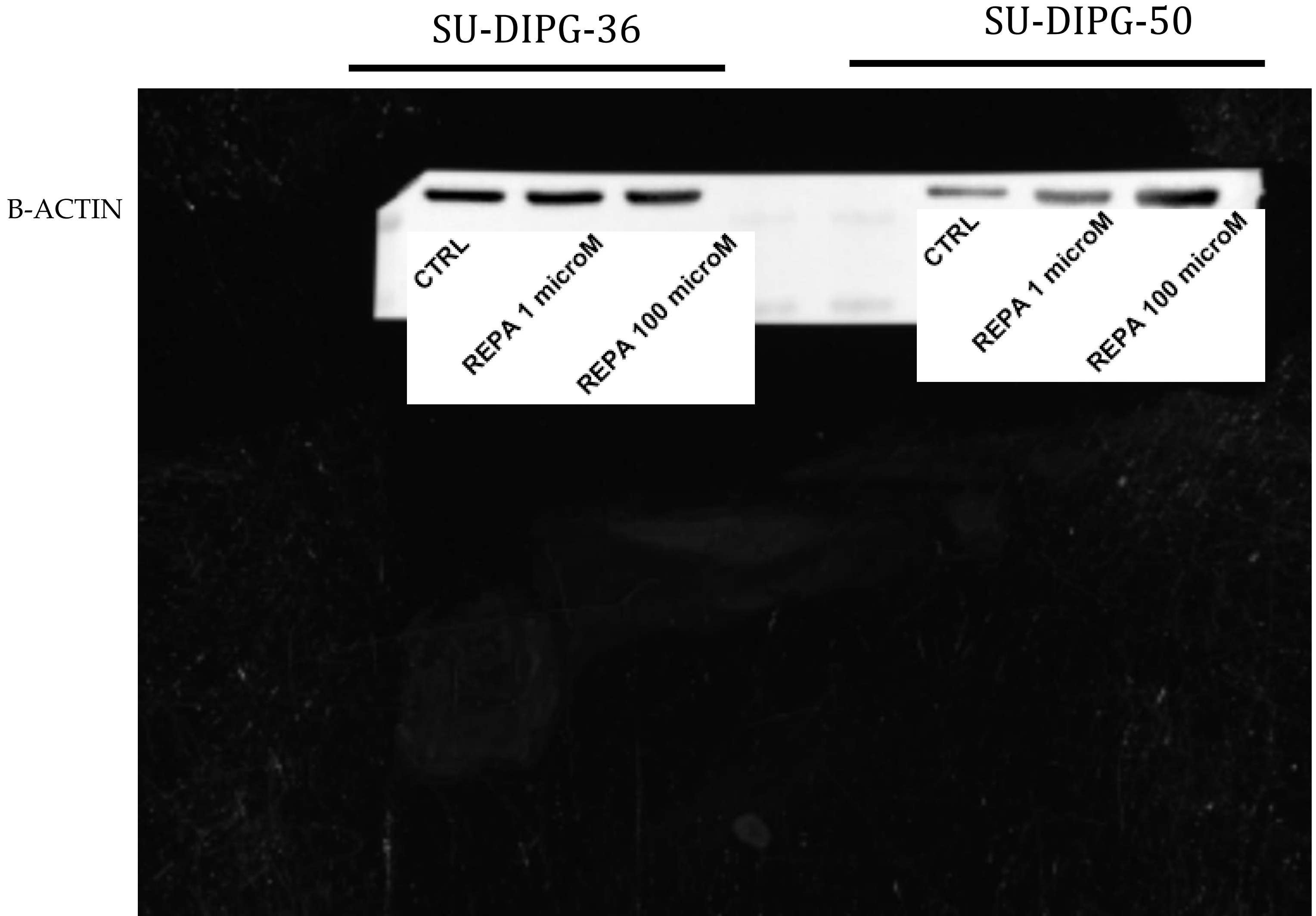
SU-DIPG-36
SU-DIPG-50
B-ACTIN
CTRL
REPA 1 microM
REPA 100 microM
CTRL
REPA 1 microM
REPA 100 microM

Gel -8

P-mTOR

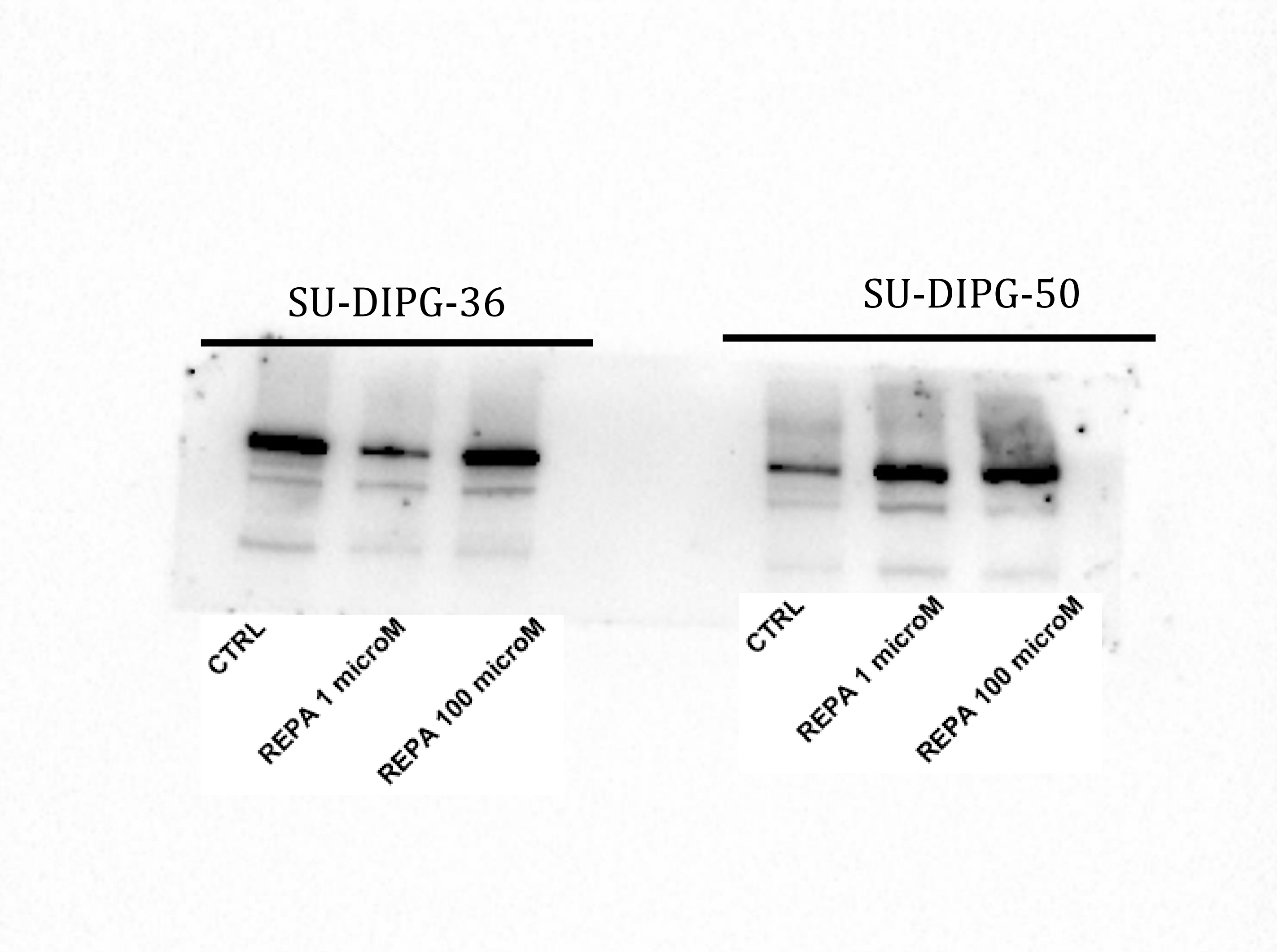

SU-DIPG-36
SU-DIPG-50
CTRL
REPA 1 microM
REPA 100 microM
CTRL
REPA 1 microM
REPA 100 microM

P-ERK 1/2

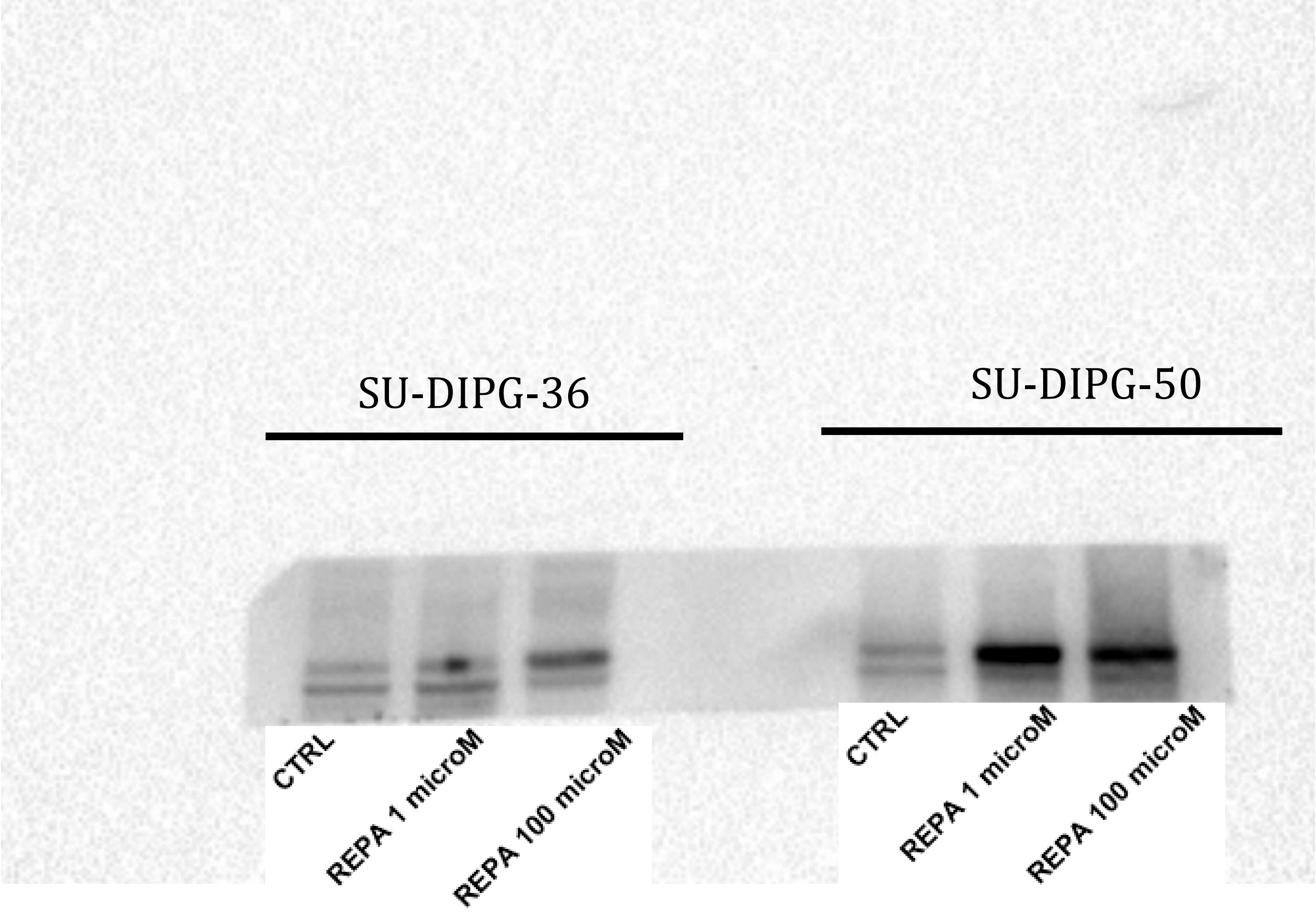
SU-DIPG-36
SU-DIPG-50
CTRL
REPA 1 microM
REPA 100 microM
CTRL
REPA 1 microM
REPA 100 microM

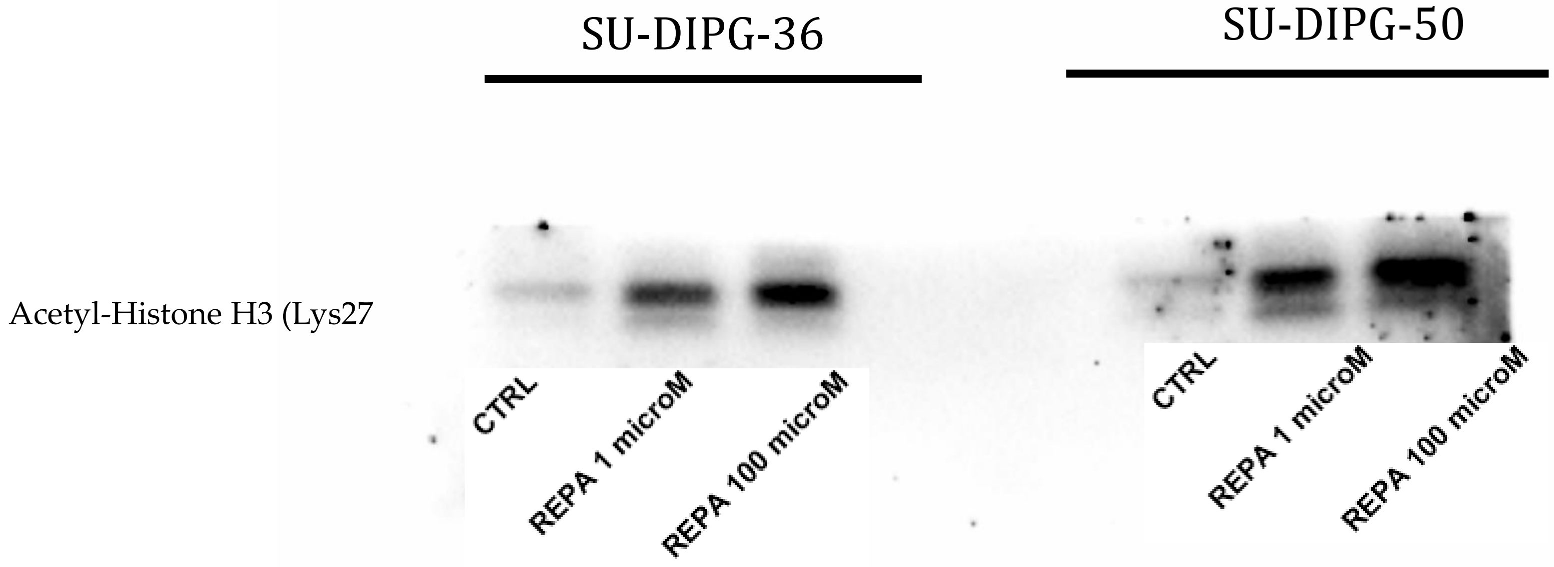
SU-DIPG-36
SU-DIPG-50
Acetyl-Histone H3 (Lys27
CTRL
REPA 1 microM
REPA 100 microM
CTRL
REPA 1 microM
REPA 100 microM

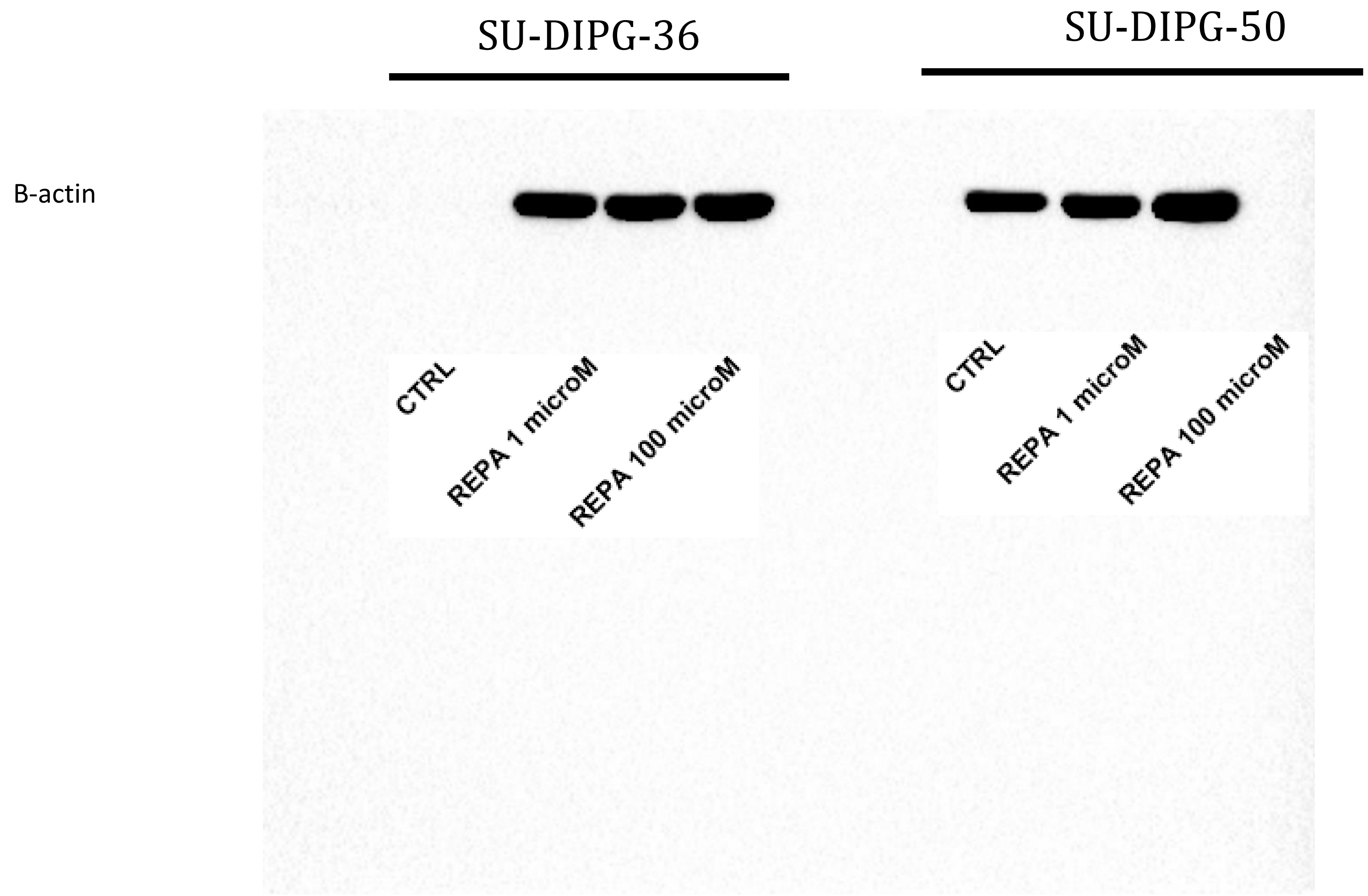

SU-DIPG-36
SU-DIPG-50
B-actin
CTRL
REPA 1 microM
REPA 100 microM
CTRL
REPA 1 microM
REPA 100 microM

Gel -9

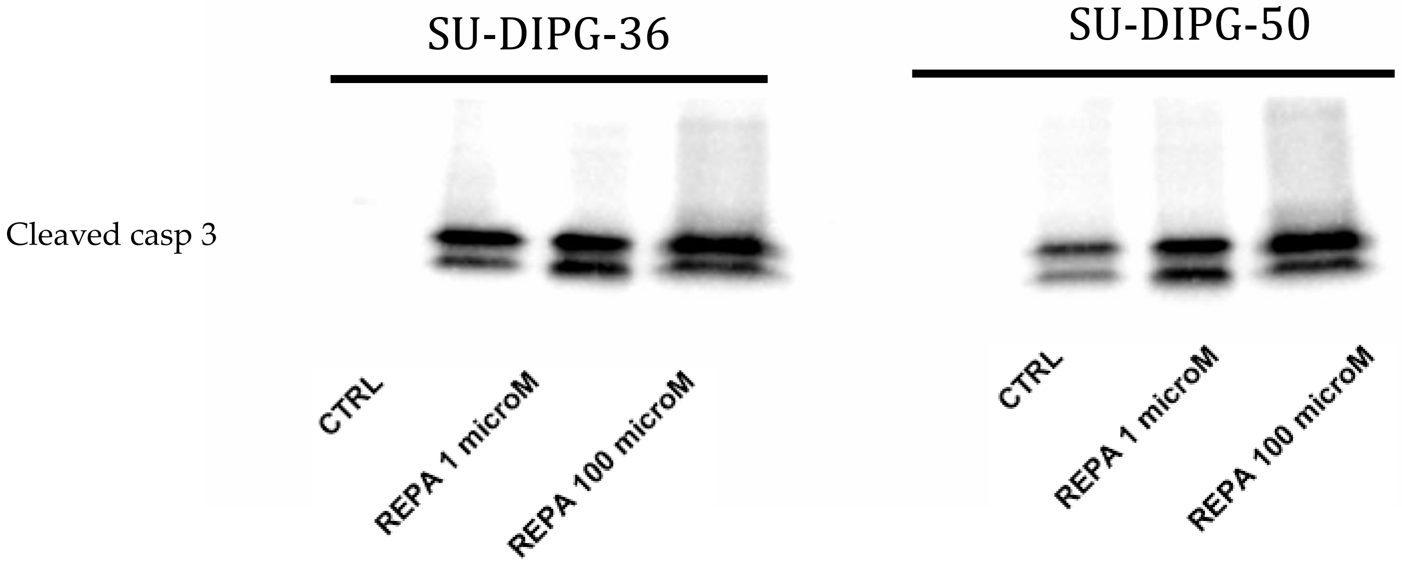

SU-DIPG-36
SU-DIPG-50
Cleaved casp 3
CTRL
REPA 1 microM
REPA 100 microM
CTRL
REPA 1 microM
REPA 100 microM

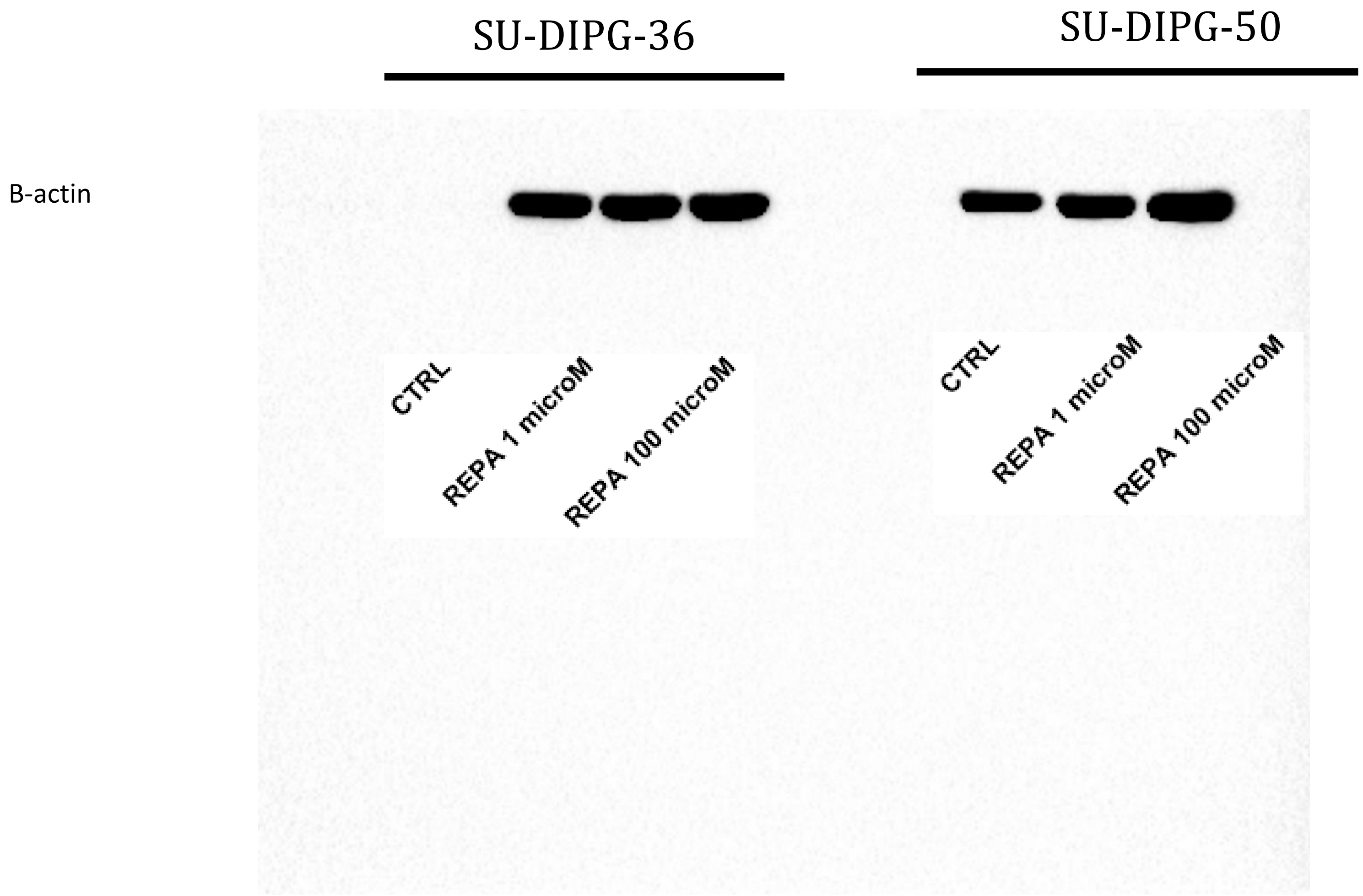
SU-DIPG-36
SU-DIPG-50
B-actin
CTRL
REPA 1 microM
REPA 100 microM
CTRL
REPA 1 microM
REPA 100 microM
